# Controlling transferrin receptor trafficking with GPI-valence in bloodstream stage African trypanosomes

**DOI:** 10.1371/journal.ppat.1006366

**Published:** 2017-05-01

**Authors:** Calvin Tiengwe, Peter J. Bush, James D. Bangs

**Affiliations:** 1 Department of Microbiology & Immunology, School of Medicine and Biomedical Sciences, University at Buffalo (SUNY), Buffalo, New York, United States of America; 2 South Campus Instrument Center, School of Dental Medicine, University at Buffalo (SUNY), Buffalo, New York, United States of America; University of California, Los Angeles, UNITED STATES

## Abstract

Bloodstream-form African trypanosomes encode two structurally related glycosylphosphatidylinositol (GPI)-anchored proteins that are critical virulence factors, variant surface glycoprotein (VSG) for antigenic variation and transferrin receptor (TfR) for iron acquisition. Both are transcribed from the active telomeric expression site. VSG is a GPI^2^ homodimer; TfR is a GPI^1^ heterodimer of GPI-anchored ESAG6 and ESAG7. GPI-valence correlates with secretory progression and fate in bloodstream trypanosomes: VSG (GPI^2^) is a surface protein; truncated VSG (GPI^0^) is degraded in the lysosome; and native TfR (GPI^1^) localizes in the flagellar pocket. Tf:Fe starvation results in up-regulation and redistribution of TfR to the plasma membrane suggesting a saturable mechanism for flagellar pocket retention. However, because such surface TfR is non-functional for ligand binding we proposed that it represents GPI^2^ ESAG6 homodimers that are unable to bind transferrin—thereby mimicking native VSG. We now exploit a novel RNAi system for simultaneous lethal silencing of all native TfR subunits and exclusive in-situ expression of RNAi-resistant TfR variants with valences of GPI^0–2^. Our results conform to the valence model: GPI^0^ ESAG7 homodimers traffick to the lysosome and GPI^2^ ESAG6 homodimers to the cell surface. However, when expressed alone ESAG6 is up-regulated ~7-fold, leaving the issue of saturable retention in the flagellar pocket in question. Therefore, we created an RNAi-resistant GPI^2^ TfR heterodimer by fusing the C-terminal domain of ESAG6 to ESAG7. Co-expression with ESAG6 generates a functional heterodimeric GPI^2^ TfR that restores Tf uptake and cell viability, and localizes to the cell surface, without overexpression. These results resolve the longstanding issue of TfR trafficking under over-expression and confirm GPI valence as a critical determinant of intracellular sorting in trypanosomes.

## Author summary

African trypanosomes, protozoan parasites that cause African Sleeping Sickness, have two structurally related secretory proteins that are critical for their success as pathogens: variant surface glycoprotein (VSG), which is responsible for evasion of host immune responses, and transferrin receptor (TfR), which is responsible for acquisition of essential iron for nutritional purposes. Both are dimers and both are attached to cell membranes by glycolipid anchors. VSG has two anchors and is found on the outer plasma membrane; TfR has just one anchor and is found in the flagellar pocket, a small restricted invagination of the plasma membrane that is the portal for transport in and out of the cell. These locations are critical to the function of each protein. To test the hypothesis that number of anchors, or valence, controls the localization of these proteins we have genetically engineered a trypanosome cell line that allows controlled expression of TfR with 0, 1, or 2 glycolipid anchors. Detailed studies of the localization and intracellular trafficking of these reporters confirm that glycolipid valence controls ultimate localization, and thus is critical to the essential functions of both VSG and TfR.

## Introduction

Many eukaryotic secretory proteins such as surface antigens, adhesion proteins, and receptors are attached to the external leaflet of the plasma membrane by glycosylphosphatidylinositol (GPI) anchors [1, 2]. GPI anchors function as vesicular transport signals for ER export, for post-Golgi sorting, and for subsequent delivery to the plasma membrane. For example, in yeast, inhibition of GPI attachment leads to delayed ER exit of the major GPI-AP, Gas1p [3]; and GPI-APs exit the ER in cargo vesicles that are distinct from other secretory and plasma membrane proteins [4]. In mammalian cells, GPI-anchors serve as cell surface targeting signals by specific association with sterol/sphingolipid-rich detergent-insoluble membranes (a.k.a. lipid rafts) at the trans-Golgi network [5, 6]. Likewise at the plasma membrane, GPI-APs preferentially cluster in lipid raft microdomains [7]. Ultimately cell surface GPI-APs play critical roles in cell adhesion in fungi, inhibition of complement lysis in erythrocytes, and in defence against host immunity in parasitic protozoa like African trypanosomes [2, 8].

**Fig 1:**
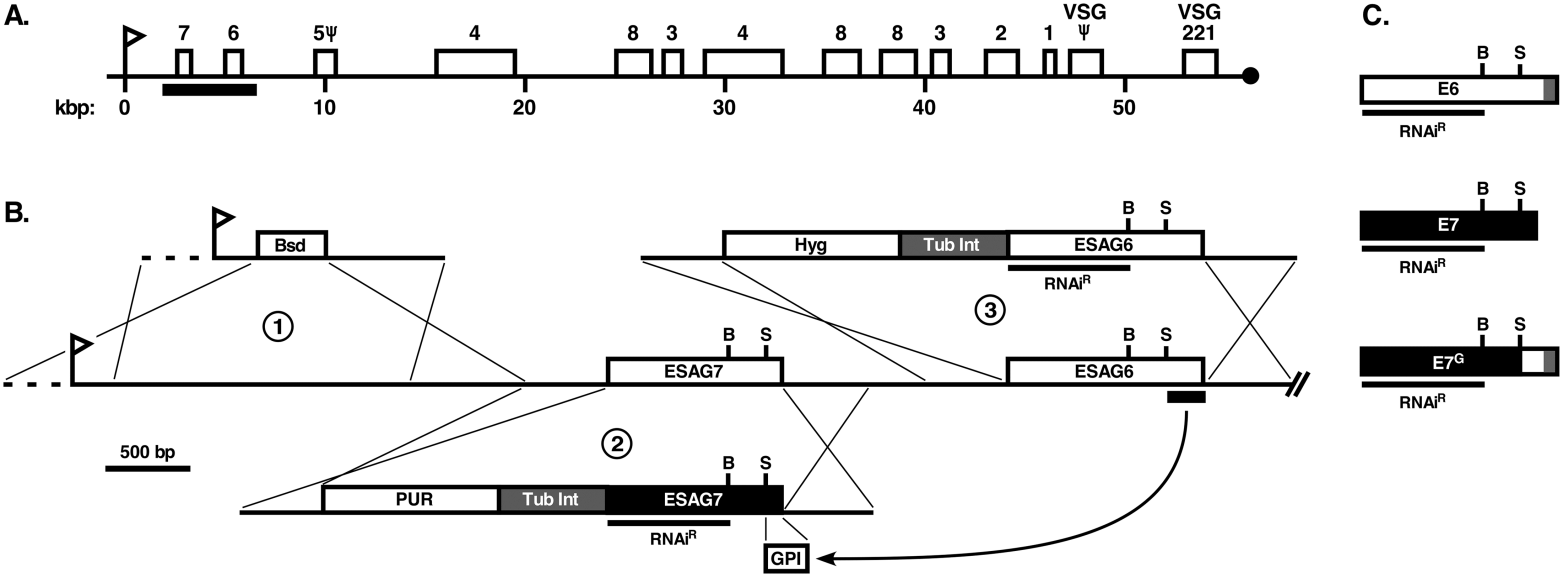
Modification of the TfR locus. **A**. Map of the active BES1 telomere showing the promoter proximal positions of ESAG6 (E6) and ESAG7 (E7) ORFs [52]. ESAG ORFs are numbered. Flag indicates the ES promoter. Under-bar indicates the region of E6 and E7 modifications. **B**. Expanded diagram of the promoter proximal region and integration constructs. (1) Blasticidin insertion cassette (Bsd). (2) RNAi^R^ E7 replacement construct containing (5’-3’): 5’ upstream targeting sequences; puromycin resistance cassette (PUR); tubulin βα-intergenic region (TubInt); RNAi^R^ E7 ORF or a fusion construct of native E7 with the C-terminal GPI signal of E6 from SacI site (S) to stop codon (GPI); 3’ downstream targeting sequences. Underbar indicates recoded RNAi^R^ region. (3) RNAi^R^ E6 replacement construct containing (5’-3’): 5’ upstream targeting sequences; hygromycin resistance cassette (Hyg); tubulin βα-intergenic region (TubInt); RNAi^R^ E6 ORF; 3’ downstream targeting sequences. Underbar indicates recoded RNAi^R^ region. Internal BamHI (B) and SacI (S) sites are indicated in both the E7 and E6 ORFs. **C**. E6 and E7 replacement ORFs. RNAi^R^ E6 and E7 ORFs, or a fusion construct of E7 with the C-terminus of E6 (E7^G^) with the C-terminus GPI signal (grey boxes); unique BamHI (B) and SacI (S) sites indicated. Under-bars indicates region of RNAi resistance (RNAi^R^).

African trypanosomes (*Trypanosoma brucei* ssp), parasitic protozoa responsible for human (Sleeping Sickness) and veterinary (nagana) trypanosomiases, have two cell surface GPI-APs that are critical to the pathogenic bloodstream (BSF) stage: variant surface glycoprotein (VSG) and transferrin receptor (TfR). VSG is a homodimer (GPI^2^) that forms a dense surface coat covering the contiguous cell body and flagellar membranes [9, 10]. It acts as a macromolecular barrier for host-derived antibodies targeting underlying invariant surface proteins. BSF trypanosomes avoid elimination by host anti-VSG immune responses by switching monoallelic expression of antigenically distinct *VSG*s from a repertoire of >1500 genes. *VSG* transcription is from a promoter distal position in a telomeric expression site (ES) (Fig 1A); there are ~15 such ESs, only one of which is active at a time [11].

TfR, which is structurally related to VSG, is a heterodimer of ESAG6 (E6) and ESAG7 (E7) (Expression Site Associated Genes). They are expressed from promoter proximal sites in the active ES (Fig 1A), but up to 20% of all TfR transcripts come from background transcription of the other ‘silent’ ESs [12]. E6 and E7 are highly similar from N-termini to the C-terminus of E7, but E6 is longer and has a C-terminal GPI attachment peptide [13, 14]. Native TfR is thus a GPI^1^ protein. In addition to functional E6:E7 heterodimers, each can form homodimers, but these cannot bind Tf [13]. At steady state TfR localizes in endosomal compartments and in the flagellar pocket, where it binds and internalizes holotransferrin (Tf) for iron acquisition; an essential nutrient for survival in the mammalian host [13, 15]. Internalized Tf is stripped of iron in acidic endosomes and the receptor is recycled to the flagellar pocket. Eventually TfR is degraded (*t*_1/2_ ~1.5 hr) in the lysosome [16].

Although GPIs were first characterized in trypanosomes [17], investigation of their role(s) in intracellular trafficking has lagged behind other model systems. However, studies in our laboratory indicate that GPIs are positive forward signals for ER exit in both BSF and procyclic insect stage (PCF) parasites. And in BSF cells at least, ER exit of GPI-APs is mediated by a distinct subset of COPII vesicle coat proteins [18]. GPIs are also critical for post-Golgi sorting of VSG via the flagellar pocket to the cell surface in BSF cells. [Throughout this report we will make a distinction between the flagellar pocket, a small invagination of the plasma membrane specialized for exo- and endocytic trafficking, and the contiguous outer cell surface comprised of the flagellum and cell body] Expression of VSG without the GPI addition signal (VSGΔgpi, GPI^0^ valence) leads to delayed ER exit followed by rapid mis-targeting to the lysosome and subsequent degradation (*t*_1/2_ ~45 min), and this holds for other GPI^0^ reporters as well [16, 19]. In contrast, native VSG (GPI^2^ valence) is rapidly delivered to the cell surface (*t*_1/2_ ~15 min) and is highly stable [20–23]. Any single VSG molecule is endocytosed and recycled repeatedly, turning over with a population half-life of >30 hr. Interestingly, a series of GPI^1^ reporters based on endogenous secretory proteins have phenotypes intermediate to GPI^0^ and GPI^2^ VSG, parsing between lysosomal targeting/degradation and transport to the cell surface [16]. Those reporters that do reach the cell surface are shed due to a quirk of GPI synthesis in BSF trypanosome—the penultimate GPI precursor is specifically remodeled to contain dimyristoyl (C14) glycerol, which alone is not sufficient to maintain long-term membrane association of a GPI^1^ protein [24, 25]. These findings have led us to propose that GPI valence controls progression within the secretory/endosomal system of BSF trypanosomes: GPI^2^-APs progress to dynamic cell surface expression; GPI^1^-APs have transient endosomal/flagellar pocket localization with ultimate parsing between lysosomal turnover and surface shedding; and GPI^0^-APs traffick by default to the lysosome for degradation.

Native TfR is unusual in this regard. At normal expression levels it is located in endosomes and the flagellar pocket, but is barely detectable in shed extracellular fractions indicating that it rarely escapes onto the cell surface. However, under conditions that BSF cells perceive as iron starvation, including altered transferrin source and hypoxia, TfR expression is dramatically upregulated and receptor is readily detectable on the cell surface, suggesting a saturable retention mechanism in the flagellar pocket [26, 27]. However, we found that upregulated surface receptor is not functional for Tf binding, nor is it shed from cells, as would be expected for a normal GPI^1^ TfR heterodimer [16]. Based on these findings, and in concordance with the GPI valence concept, we proposed that surface TfR actually represents GPI^2^ homodimers of E6, which would be expected to behave essentially as homodimeric VSG.

In this study we further explore these alternative possibilities, and in so doing challenge the GPI valence model. Using an RNAi cell line to eliminate background expression of TfR from silent ESs [28], we have engineered in situ expression of RNAi resistant (RNAi^R^) versions of wild type and modified E6 and E7 subunits from the active ES. This functional complementation of RNAi approach [29] allows controlled analyses of the trafficking, localization, and turnover of TfRs of GPI^0–2^ valence. Our results are fully consistent with the valence hypothesis and provide strong supportive evidence for our model for surface localization of over-expressed TfR.

## Results

### TfR silencing and functional complementation by RNAi^R^ E6 and E7

**Fig 2:**
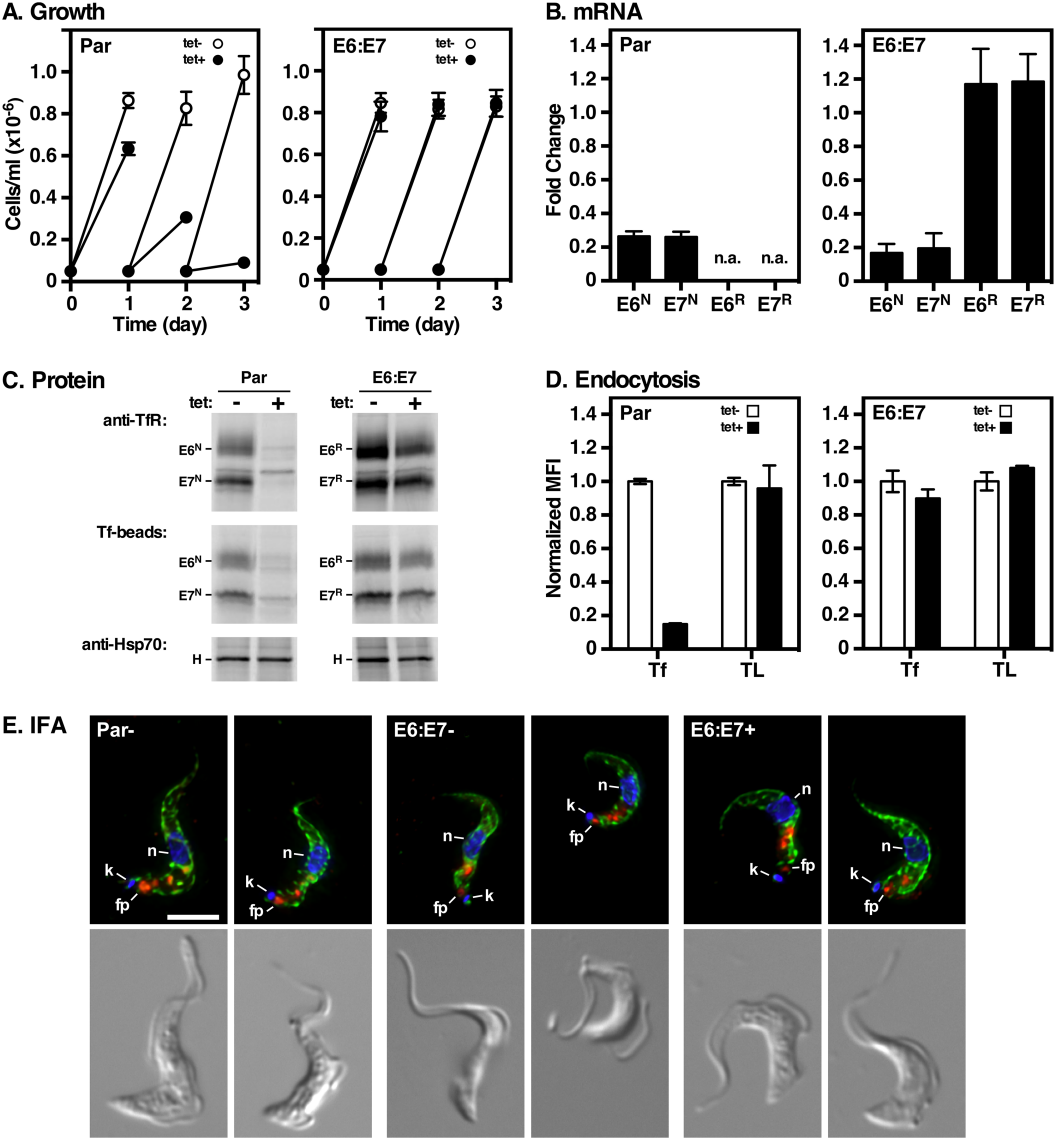
TfR RNAi and functional complementation. The parental TfR RNAi cell line alone (Par), or complemented with RNAi^R^ E6^R^ and E7^R^ constructs (E6:E7), were cultured without (tet-) or with tetracycline (tet+). **A**. Cell density was measured by hemocytometer and cultures were adjusted to starting density daily. Data are means ± SEM (n = 3). All subsequent analyses were performed at 24 hrs of silencing. **B**. Total RNA was prepared from the Par or E6:E7 cell lines. Transcript levels of native RNAi-sensitive E6^N^ and E7^N^ and RNAi-resistant E6^R^ and E7^R^ were determined by qRT-PCR; n.a indicates not assayed. Specific primers are indicated in S1 Fig. Results are normalized to un-induced controls and are presented as fold-change for three biological replicates (mean ± SEM). C. Un-induced and induced Par and E6:E7 TfR RNAi cells were pulse radiolabeled (1 hr) with [^35^S]Met/Cys and polypeptides were specifically pull-downed with rabbit anti-TfR antibodies (anti-TfR), transferrin-conjugated beads (Tf-beads) or rabbit anti-HSP70 antibodies (anti-HSP70). Pull-downs were fractionated by SDS-PAGE (10^7^ cell equivalents/lane) and visualized by phosphorimaging. The mobilities of ESAG6 (E6), ESAG7 (E7) and HSP70 (H) are indicated on the left of the appropriate panels. Representative phosphorimages are presented (n = 3). **D**. Receptor mediated uptake of fluorescent transferrin (Tf) and tomato lectin (TL) was measured by flow cytometry. Data are presented as median fluorescent intensity (MFI ± SEM.) for three biological replicates and are normalized to un-silenced control cells. **E**. Localization of native TfR in cells without (Par-, E6:E7-) or with (E6:E7+) tetracycline. Cells were fixed, permeabilized and stained with mouse anti-BiP (green), rabbit anti-TfR (red), and DAPI (blue) to detect nucleus (n) and kinetoplast (k). In each case flagellar pocket localizations of TfR are indicated (fp). Deconvolved three-channel summed stack projections of representative cells (top panel) with the matched DIC images (bottom panel) are presented. Bar = 4 μm.

Controlled genetic manipulation of TfR genes has been impossible because ~20% of TfR transcripts derive from ‘silent’ ESs [12]. Therefore, to study the behavior of TfR subunits without background interference, we created a parental RNAi cell line targeting all native TfR transcripts (both E6 and E7) regardless of source [28]. The native *E6* and *E7* ORFs (*E6*^*N*^ and *E7*^*N*^) in the active ES were then replaced with recoded RNAi^R^ ORFs (*E6*^*R*^ and *E7*^*R*^) taking care to preserve the native 3’ UTRs so that normal expression levels remained unaltered (Fig 1B). As previously reported [28], TfR silencing was lethal over a period of 3 days (Fig 2A, Par), although cells remained viable after 24 hours of induction due to excess iron stores [30, 31]. The TfR RNAi-mediated growth phenotype was completely rescued by co-expression of both *E6*^R^ and *E7*^R^ genes from the active ES (Fig 2A, E6:E7). All subsequent analyses were performed at 24 hours post-silencing since cells remained viable with excellent morphology at this time point.

TfR silencing led to specific depletion (~80%) of *E6*^*N*^ and *E7*^*N*^ transcripts in both parental (Fig 2B, left) and RNAi resistant E6^R^:E7^R^ cell lines (Fig 2B, right), while *E6*^*R*^ and *E7*^*R*^ transcripts remained unaffected in E6^R^:E7^R^ cells (Fig 2B, right). Pull-down experiments with metabolically labeled cells were performed to assess the effect of silencing on TfR biosynthesis. Stoichiometric amounts of metabolically labeled E6 and E7 were captured by pull down with anti-TfR and Tf ligand in both parental and E6^R^:E7^R^ cells (Fig 2C, tet-). Induction of dsRNA almost completely eliminated E6 and E7 synthesis in the parental cells (Fig 2C, left), but subunit synthesis was unaffected in E6^R^:E7^R^ cells (Fig 2C, right). Loss of TfR predictably abolished Tf uptake in parental cells (Fig 2D, left), but was restored to wild-type levels in the RNAi^R^ cells (Fig 2D, right). In each case uptake of tomato lectin (TL), a surrogate for receptor-mediated endocytosis [32, 33], was normal confirming that general endocytosis was unaffected. Finally, native TfR localizes normally to the flagellar pocket and endocytic compartments (Fig 2E, Par, red). In the absence or presence of native TfR silencing, a similar TfR staining pattern was seen in RNAi^R^ cells (Fig 2E, E6:E7, red) indicating that trafficking of E6^R^:E7^R^ TfR is normal. These results show that co-expressed E6^R^ and E7^R^ form functional TfR heterodimers, which was confirmed by BN-PAGE (S2 Fig). Taken together these findings fully validate our experimental system. Subsequent experiments involve expression of E7^R^, E6^R^ and E7^G^ (Fig 1C) alone or in combination in the TfR RNAi cell line.

### Expression of E7^R^ (GPI^0^)

**Fig 3:**
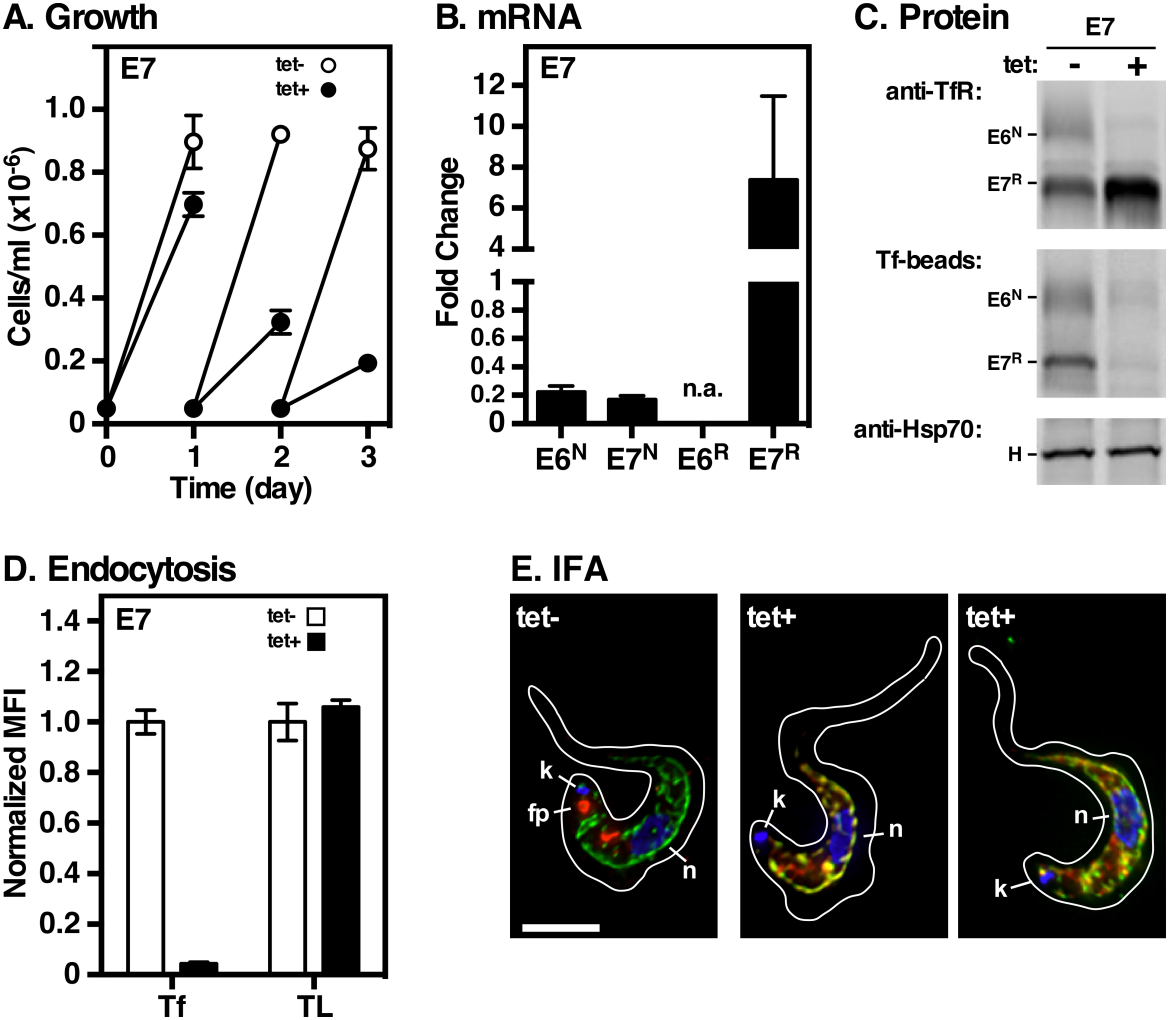
Expression and function of E7^R^ alone. The parental TfR RNAi cell line containing RNAi resistant E7^R^ was cultured without (tet-) or with (tet+) tetracycline. **A**. Cell density was measured as in Fig 2. Data are means ± SEM (n = 3). All subsequent analyses were performed at 24 hrs of silencing. **B**. Levels of native E6^N^, E7^N^ and RNAi^R^ E7^R^ transcripts were determined by qRT-PCR as in Fig 2; n.a indicates not assayed. Results are normalized to un-induced controls and are presented as fold-change for three biological replicates (mean ± SEM.). **C**. E7 cells were pulse radiolabeled as in Fig 2, and pull-downs performed with anti-TfR, Tf-beads, and anti-HSP70. The mobilities of E6, E7 and HSP70 (H) are indicated. All images are representative of three independent biological replicates. **D**. Receptor mediated uptake of fluorescent transferrin (Tf) and tomato lectin (TL) was measured by flow cytometry. Data are presented as median fluorescent intensity (MFI ± SEM.) for three biological replicates and are normalized to un-silenced control cells. **E**. IFA of the E7^R^ cell line without (tet-) or with (tet+) tetracycline as in Fig 2 with anti-BiP (green), anti-TfR (red), and DAPI (blue) to detect nucleus (n) and kinetoplast (k). As appropriate, flagellar pocket localization of TfR is indicated (fp). Deconvolved three-channel summed stack projections of one (tet-) or two (tet+) representative cells. Cell outlines were traced from matched transmitted light images. Bar = 4 μm.

*E7*^*R*^ was inserted into BES1 leaving the native *E6* gene intact. Without silencing, growth was normal indicating formation of functional E6^N^:E7^R^ heterodimers (Fig 3A), which was confirmed by pull down experiments with Tf-beads (Fig 3C) and uptake experiments (Fig 3D). However, when native E6 was ablated by RNAi (Fig 3B, ~80%) E7^R^ alone was insufficient to maintain cell growth. This correlated with complete loss of E6 synthesis (Fig 3C), and of Tf binding (Fig 3C) and endocytosis (Fig 3D). Interestingly, in the absence of E6 there was a marked up-regulation in steady-state *E7*^*R*^ transcripts (~8 fold) and in E7^R^ synthesis. We interpret this phenomenon as an iron starvation response in the absence of functional TfR, similar to the up-regulation of TfR observed when cells are deliberately starved for transferrin [16, 26, 27]. In control cells, TfR localization was identical to functional native TfR—endosomal and flagellar pocket (Fig 3E, tet-, red). In contrast, the TfR signal (E7^R^ only) dramatically increased in silenced cells, and overlapped markedly with BiP, consistent with ER localization (Fig 3E, tet+, yellow). ER accumulation could be due to the absence of GPI anchors as forward ER exit signals on E7^R^ homodimers [18, 19], and/or to improper folding/dimerization [28]. BN-PAGE indicates that E7^R^ is present primarily as dimers with a significant amount of low mobility smearing consistent with both possibilities (S2 Fig). Overall these results are in general agreement with the valence model.

### Expression of E6^R^ (GPI^2^)

**Fig 4:**
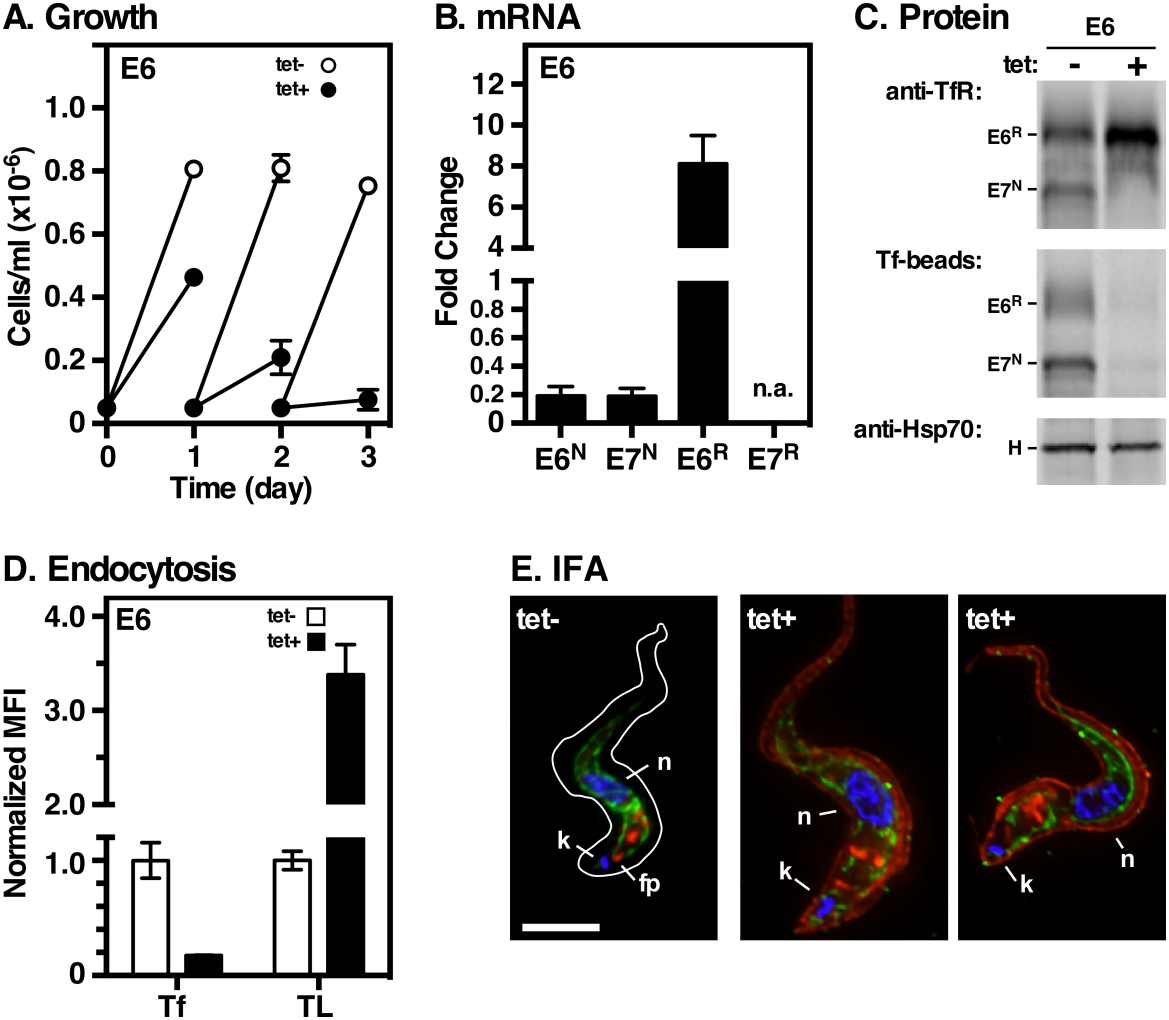
Expression and function of E6^R^ alone. The parental TfR RNAi cell line containing RNAi resistant E6^R^ was cultured without (tet-) or with (tet+) tetracycline. All analyses are identical to Fig 3. **A**. Cell density. **B**. Transcript levels by qRT-PCR. **C**. Biosynthesis and pull-down of TfR subunits. All phosphorimages are representative of three independent biological replicates. **D**. Receptor mediated endocytosis by flow cytometry. **E**. IFA of fixed permeabilized cells with mouse anti-BiP (green), rabbit anti-TfR (red), and DAPI (blue) to detect nucleus and kinetoplast. Cell outline (tet- only) was traced from matched transmitted light images. Deconvolved three-channel summed stack projections of representative cells are shown. Bar = 4 μm.

*E6*^R^ alone was inserted into the active expression site of TfR RNAi cells, and all phenotypic analyses were performed as described above for E7^R^ cells. Without silencing, E6^R^ cells grew normally (Fig 4A), and functional E6^R^/E7^N^ heterodimers were detected by Tf pull-down (Fig 4C) and uptake (Fig 4D) assays. TfR silencing ablated all native *E6* and *E7* transcripts (Fig 4B) and synthesis of native E7 subunit (Fig 4C). Depletion of E7^N^ also resulted in up-regulation of *E6*^*R*^ transcript levels (~8-fold) and synthesis. As with the discrete expression of E7^R^, we interpret this as a response to perceived iron starvation. TfR (E6^R^ only) was still localized in endosomal compartments after silencing of E7^N^, but a prominent signal of surface and flagellar staining became apparent (Fig 4E, tet+). RNAi-dependent surface expression was confirmed by flow cytometry of non-permeablized cells (S3 Fig). Interestingly there was a 3-fold increase in uptake of tomato lectin in TfR silenced cells (Fig 4D, TL). We attribute this increase to elevated expression and surface localization of the E6^R^ protein, which is known to have glycan epitopes reactive with this lectin [34, 35]. Finally, BN-PAGE confirms that E6^R^ forms GPI^2^ homodimers in TfR silenced cells (S2 Fig). Collectively these data, homodimerization and surface expression, are fully consistent with the valence hypothesis. However, because surface expression was only detected under conditions of E6^R^ up-regulation, we cannot completely eliminate a saturable retention mechanism for exclusion of TfR from the cell surface.

### Expression of E6^R^:E7^G^ (GPI^2^)

**Fig 5:**
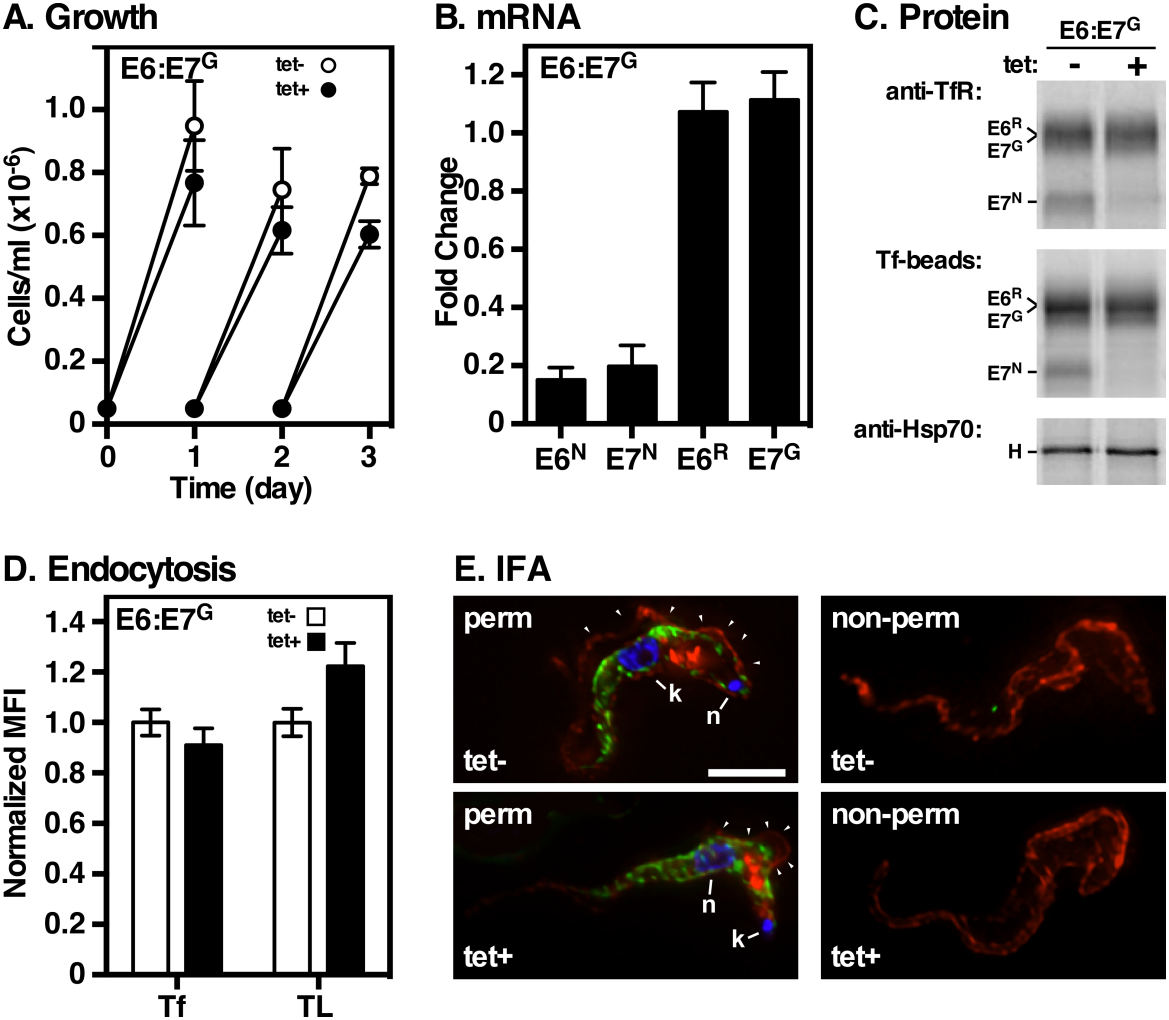
Expression and function of E6^R^:E7^G^. The parental TfR RNAi cell line containing RNAi resistant E6^R^ and E7^G^ (E6^R^:E7^G^) was cultured without (tet-) or with (tet+) tetracycline. All analyses are identical to Fig 3. **A**. Cell density. **B**. Transcript levels by qRT-PCR. **C**. Biosynthesis and pull-down of TfR subunits. Note that the E6^R^ and E7^G^ ORFs/proteins are the same length/size. All phosphorimages are representative of three independent biological replicates. **D**. Receptor mediated endocytosis by flow cytometry. **E**. IFA of fixed permeabilized and non-permeabilized cells, as indicated. Permeable cells (left) were stained with anti-BiP (green), rabbit anti-TfR (red), and DAPI (blue) to detect nucleus and kinetoplast. Arrowheads indicate surface staining along the flagellar membrane. Non-permeable cells (right) were stained with anti-TfR alone. Deconvolved three-channel summed stack projections of representative cells (tet-) or (tet+) are shown. Bar = 4 μm.

In order to rule out up-regulation as a confounding factor, a cell line expressing functional GPI^2^ TfR was generated. The C-terminus of E6, with GPI attachment signal, was fused to E7 (E7^G^), and this construct was co-expressed with E6^R^ generating the RNAi^R^ E6^R^:E7^G^ cell line. Growth was normal under TfR silencing (Fig 5A), suggesting formation of functional GPI^2^ heterodimers. qRT-PCR and pull down analyses confirmed loss of *E6*^*N*^/*E7*^*N*^ transcripts (~80%), and of E7^N^ protein. However, *E6*^*R*^ and *E7*^*G*^ transcripts, and corresponding protein levels, were unaffected, (Fig 5B and 5C respectively), suggesting that cells were not iron deprived. In agreement, Tf binding (Fig 5C) and uptake (Fig 5D; Tf) were unaffected. The formation of GPI^2^ heterodimers was confirmed by BN-PAGE (S2 Fig). Finally, in addition to endosomal localization, IFA in both control and silenced cells showed low intensity cell surface TfR staining in permeabilized cells (Fig 5E, perm), and more prominently in non-permeabilized cells (Fig 5E, non-perm). Surface localization was confirmed by flow cytometry (S3 Fig). These results indicate that E6^R^:E7^G^ form functional GPI^2^ heterodimers that rescue growth, mediate Tf binding and uptake, and localize to the cell surface. Most importantly, surface accumulation occurs without over-expression, arguing against a saturable retention mechanism and demonstrating that GPI^2^ valence can override any restrictions on TfR trafficking that may be imposed by flagellar pocket architecture. These results are fully consistent with the GPI valence model.

### Trafficking and turnover kinetics of TfRs

**Fig 6:**
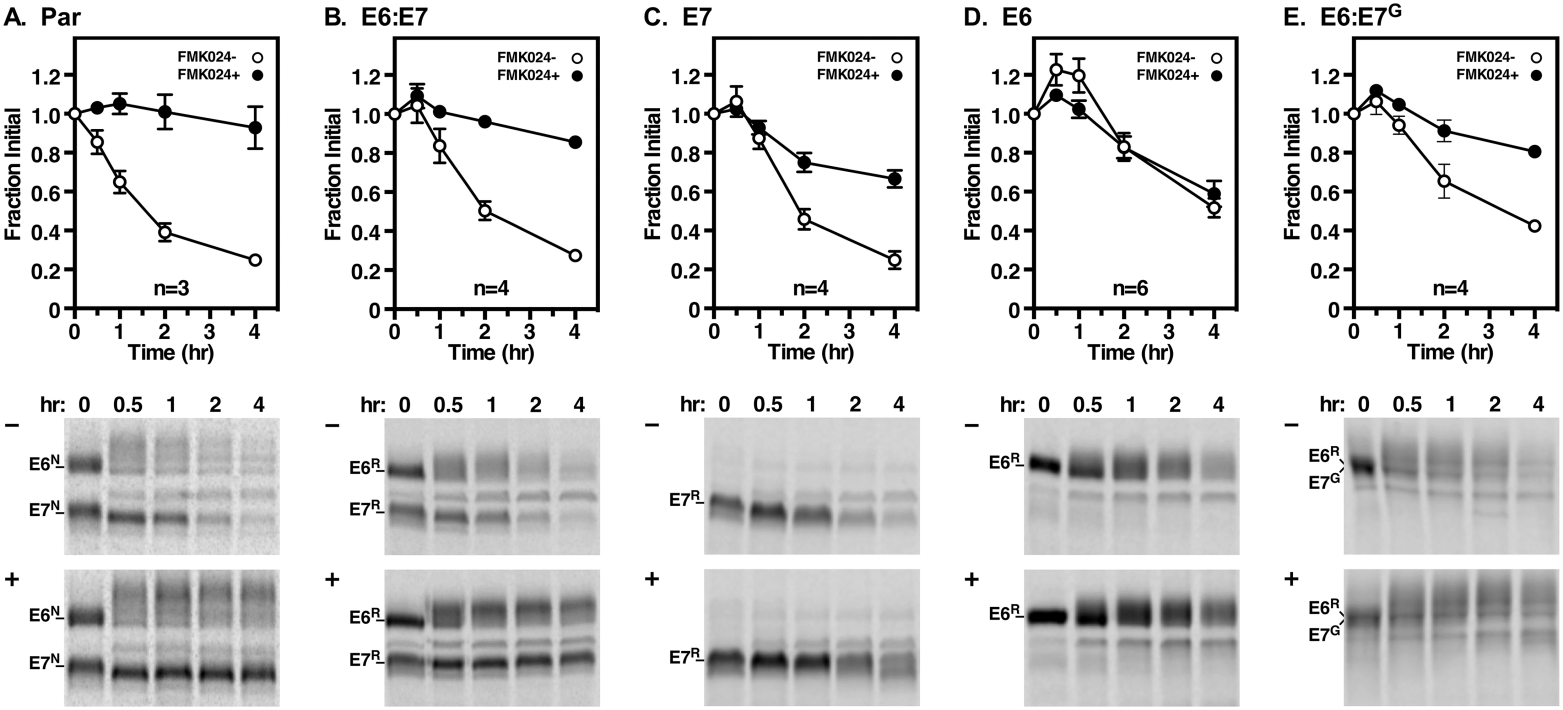
Turnover of native and RNAi^R^ TfR. Cell lines as indicated were cultured (24 hr) without (**A**. Par) or with tetracycline for all other cell lines (**B-E**). Cells were pulse/chase radiolabeled (15 min/4 hr) in the absence (-, open circles) or presence (+, closed circles) of FMK024 (20 μM) to block lysosomal degradation. At the indicated times E6 and E7 polypeptides were immunoprecipitated from cell lysates with anti-TfR antibody and fractionated by SDS-PAGE (5x10^6^ cell equivalents/lane). The rates of turnover were quantified from phosphorimages as fraction of initial species (mean ± SEM) for multiple biological replicates (n values are inset in each graph). For Par (**A**) and E6:E7 (**B**) quantifications combined E6 and E7 values are presented, but identical results were obtained when quantified individually. Representative phosphorimages are presented below each corresponding decay curve. Mobilities of E6 and E7 subunits are indicated on the left and chase times (hr) are shown above each lane.

We first confirmed that the E6^R^ and E6^R^:E7^G^ dimers were indeed GPI anchored using reactivity with anti-Cross Reacting Determinant (CRD) antibodies following GPI hydrolysis by endogenous GPI-phospholipase C (S4 Fig). We then investigated the ultimate fates of the various RNAi^R^ TfRs by quantitative turnover analyses (Fig 6). In agreement with our previous work [16], normal TfR heterodimer turns over with a half-life of ~1.5 hr in both the parental (Fig 6A) and RNAi resistant E6^R^:E7^R^ cell lines (Fig 6B). In each case loss of both E6 and E7 subunits is completely rescued by treatment with the lysosomal cathepsin L (TbCatL) inhibitor, FMK024. Inhibition revealed accumulation of E6 as a larger mature form presumably due to glycan processing during transit of the Golgi. The E7 cell line, which contains both homodimers and aggregates (S2 Fig) presents a more complex decay profile (Fig 6C). The apparent overall loss rate in untreated cells is similar to that of normal TfR heterodimers, but is only ~70% rescued by FMK024, representing lysosomal degradation of GPI^0^ homodimers. The remaining portion could represent turnover by ER-associated degradation (ERAD), but it cannot be rescued with the proteasomal inhibitor MG132, as would be expected for misfolded secretory proteins in trypanosomes [28]. Turnover of GPI^2^ E6^R^ homodimers was markedly delayed relative to normal TfR (*t*_1/2_ ~4 hr), but was unaffected by inhibition of TbCatL (Fig 6D). MG132 also had no effect, nor was E6 detected in the media fraction during the chase period. Turnover of GPI^2^ E6^R^:E7^G^ was also delayed (~2-fold) relative to normal TfR, but unlike E6 homodimers, was more fully rescued by FMK024 indicating lysosomal degradation. Generally these results are consistent with a correlation of increased GPI valence with increased stability—with two caveats. First, as there are aggregates in E7^R^ cells (S2 Fig), and as degradation is not completely rescued by FMK024, we cannot be certain of the true turnover rate for bona fide GPI^0^ E7 homodimers. Second, we can offer no explanation for the actual mode of turnover of GPI^2^ E6 homodimers, and why it differs from that of GPI^2^ E6^R^:E7^G^ heterodimers. Finally it is worth noting that while more stable than GPI^0^ or GPI^1^ TfRs, both GPI^2^ TfRs are still much less stable than native VSG (*t*_1/2_ >30 hr; discussed below).

### Surface E6^R^:E7^G^ is functional

**Fig 7:**
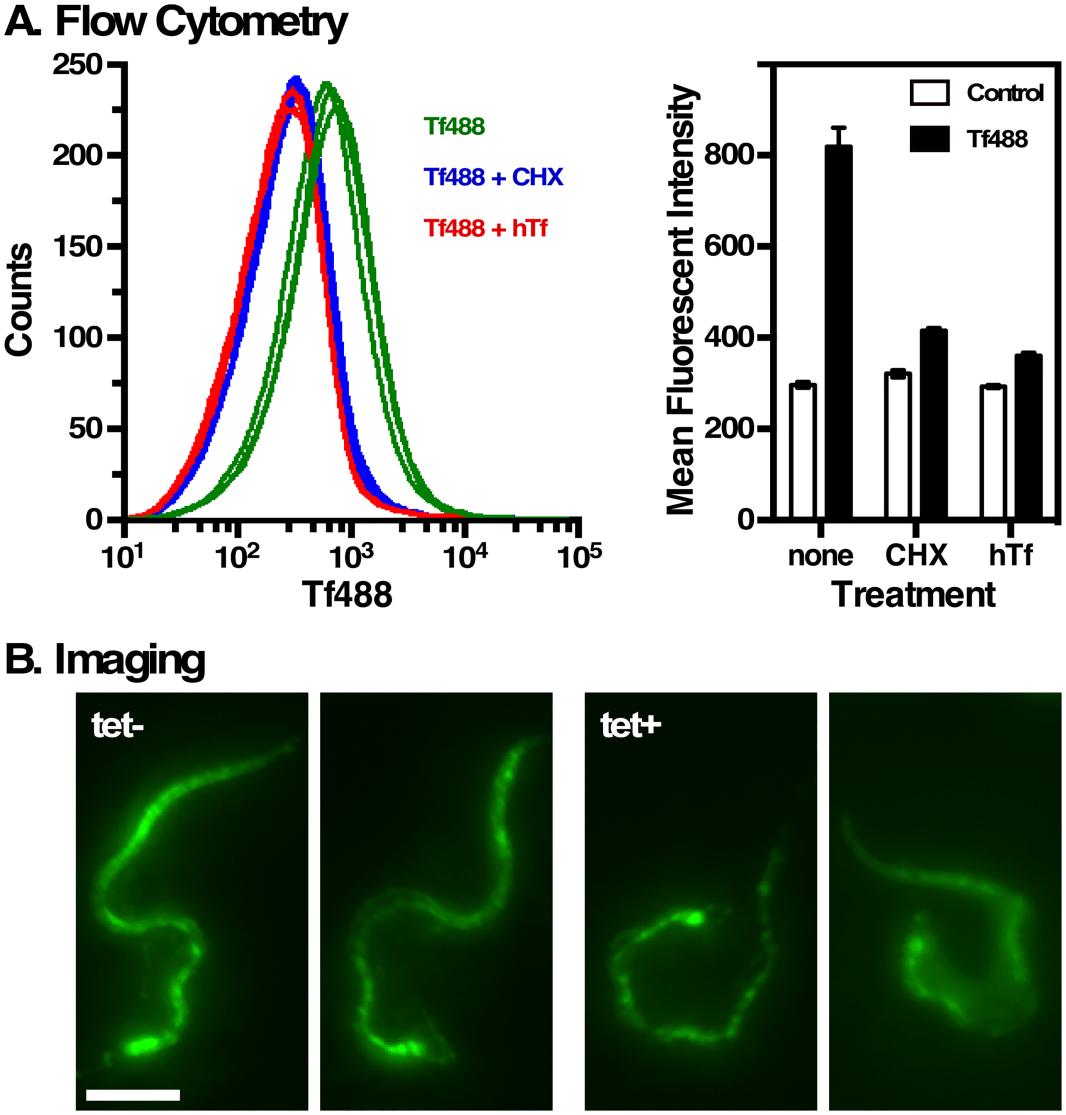
Functional localization of E6^R^:E7^G^. **A**. Silenced E6^R^:E7^G^ cells were preincubated (2 hr, 37°C) in serum free media to allow turnover and replacement of pre-existing ligated TfR with new unligated receptor. Cycloheximide (CHX) was included as indicated to block synthesis of new TfR. Alexa488-Tf (Tf488) labeling was performed with live cells as described in Methods. Holo-transferrin (hTf) competitor was included as indicated. Flow cytometry histograms of three biological replicates (left). Mean fluorescent intensities (arbitrary units, mean ± sem, n = 3) for all conditions (right). Controls are minus Tf488. **B**. Control (tet-) and silenced (tet+) E6:E7^G^ cells were preincubated and labeled as above, and then imaged by epifluorescence. Images are identically contrast enhanced, un-deconvolved, summed stack projections.

**Fig 8:**
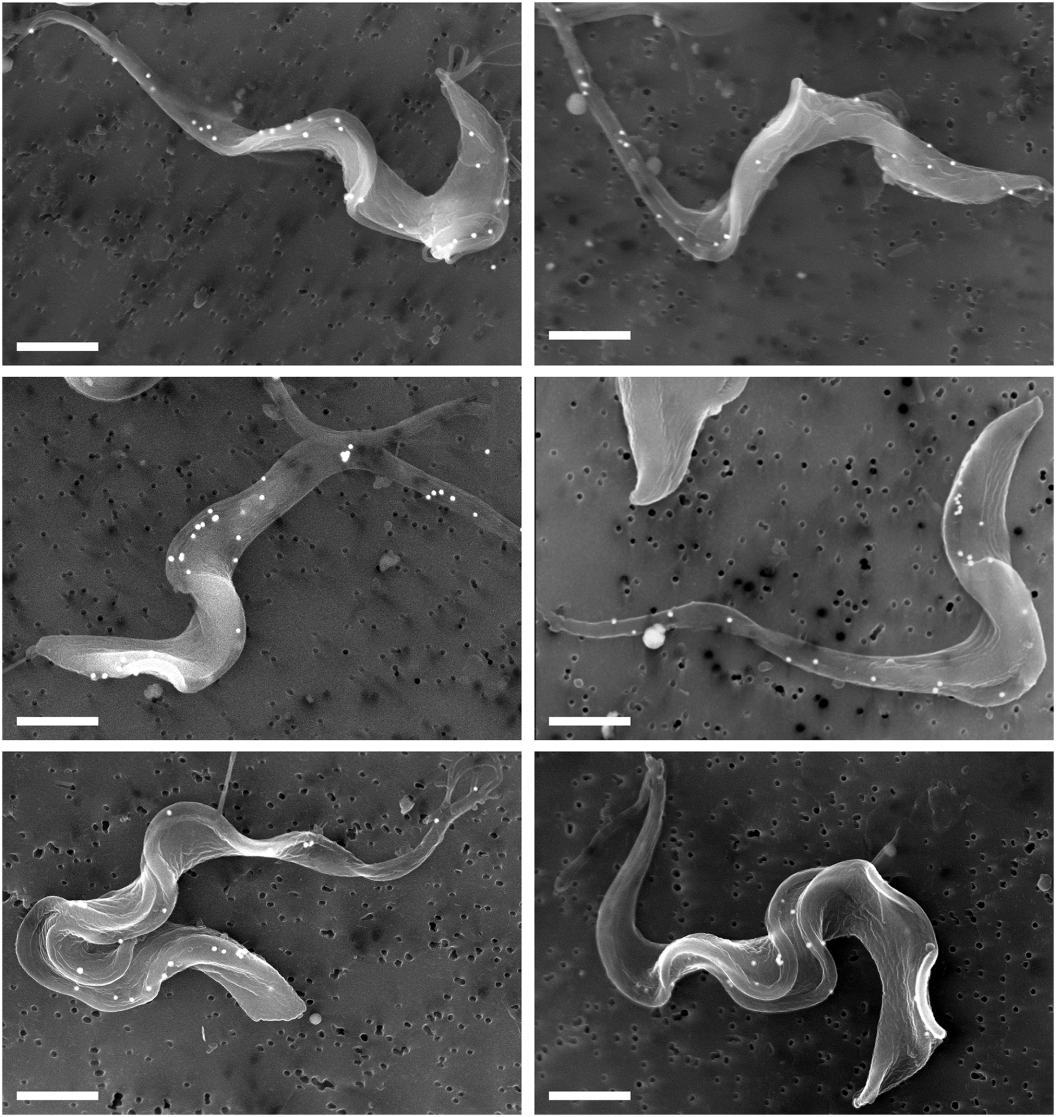
Surface localization of E6^R^:E7^G^ by scanning electron microscopy. RNAi silenced E6^R^:E7^G^ cells were pre-incubated (2 hr, 37°C) in serum free media and labeled alive (4°C) with Tf:gold (100 nm), then fixed and prepared for EM as described in Methods. White dots are bound colloidal gold particles. Bars indicate 2 μm. Images of precytokinesis cells (two flagella) are presented in the bottom panels.

The functionality of surface E6^R^:E7^G^ was investigated by assaying direct binding of fluorescent Tf. All attempts with cells freshly harvested from culture failed, presumably because surface TfR was already saturated with Tf from complete medium. However, preincubation in serum-free media to generate newly synthesized non-ligated TfR on the cell surface allowed detection of direct binding by flow cytometry (Fig 7A). Surface labeling was blocked when cycloheximide was included during the preincubation, confirming the need for ongoing protein synthesis, and binding was inhibited by excess unlabeled transferrin. Fluorescent imaging revealed prominent flagellar staining, with diffuse staining over the cell body (Fig 7B). Again binding was dependent on synthesis of new receptor and was blocked by excess Tf. Importantly, binding was observed even when native TfR subunits were ablated by RNAi silencing confirming that the signal is specific for E6^R^:E7^G^ heterodimers. To investigate at higher resolution, SEM was performed on cells that were pre-labeled with Tf-conjugated colloidal gold (Fig 8). Consistent with fluorescent imaging, gold particles were prominently detected in close proximity to the flagella and flagellar attachment zone, but also to a lesser degree over the cell body. Binding was blocked by excess transferrin, and no binding was observed in the parental RNAi cell line. These results conclusively demonstrate that cell surface E6^R^:E7^G^ heterodimer is functional for Tf binding.

## Discussion

We have investigated two interrelated aspects of GPI function in African trypanosomes, the role of GPI valence in post-Golgi sorting, and the localization of TfR at normal and elevated expression levels. GPI valence broadly correlates with secretory progression and stability [16, 19]. Native VSG (GPI^2^) is a super-abundant surface protein that is constantly endocytosed and recycled to the cell surface. It is slowly shed from cells (*t*_1/2_ >30 hr) by a combination of exocytic vesicles and GPI hydrolysis [21, 23, 36]. Any lysosomal degradation, if it does occur, is below the limits of detection. However, GPI-minus VSG, as well as other GPI^0^ reporters, are rapidly (*t*_1/2_ <1 hr) delivered to the lysosome and degraded by resident thiol proteases. In contrast, a series of GPI^1^ reporters engineered on native secretory proteins have a continuum of intermediate behaviors. When GPI anchored, the ATPase domain of the ER chaperone BiP is at one extreme, being overwhelmingly delivered to the cell surface followed by shedding into the medium [one dimyrstoylglycerol-GPI freely dissociates from membranes]. Native TfR is at the other extreme, essentially being all degraded in the lysosome [*t*_1/2_ ~1.5 hr, [16] and this work]. In between are insect stage procyclin, which parses evenly between these two fates, and the lysosomal glycoprotein p67, which is mostly delivered to the lysosome (~85%) when GPI-anchored. However, TfR is a special case in that it does escape to the cell surface when over-expressed. Originally it was proposed that escape results from saturation of a flagellar pocket retention mechanism [26, 27]. Later, because such surface TfR is not shed, as would be expected for a GPI^1^ heterodimer, and is non-functional for ligand binding, we proposed that surface TfR represents GPI^2^ E6 homodimers [16].

To challenge our valence model, and to resolve the issue of TfR surface expression, we used a novel system for exclusive expression of TfR subunits [28], the critical features of which are the conditional ablation of all native TfR transcripts, regardless of source, and the expression of recoded RNAi^R^ *E6* and *E7* genes from endogenous loci within the active expression site. Silencing completely abrogates TfR synthesis, and consequently Tf uptake and cell viability. Importantly, all essential functions are restored by co-expression of RNAi^R^ TfR subunits, fully validating our approach for independent expression of E6^R^ (GPI^2^) and E7^R^ (GPI^0^) homodimers, and the special case of E6^R^:E7^G^ (GPI^2^) heterodimer. All three behave largely as predicted by the valence model—E7 homodimers are degraded in the lysosome and E6 homodimers are delivered to the cell surface. However, in each instance these subunits are dramatically over-expressed (7–8 fold), presumably in response to perceived iron starvation in the absence of functional TfR. We have also found that E7^G^ localizes to the cell surface when expressed alone, but again with significant over-expression. Consequently, one might still argue that surface localization of GPI^2^ TfR dimers results from saturation of a flagellar pocket retention mechanism. However, the E6^R^:E7^G^ heterodimer, which is functional for Tf uptake, is not up-regulated and yet is still found on the cell surface. Collectively, these results argue compellingly for GPI valence as the critical determinant for cell surface localization of TfR when expressed at either normal or elevated levels. They do not however, *sensu stricto*, prove that GPI^2^ valence is sufficient, rather than merely necessary, to achieve surface expression as there may be other feature(s) of E6/E7 dimers that are necessary for egress from the flagellar pocket. However, this does seem unlikely given that TfR has evolved not to be surface exposed.

Although our results are broadly consistent with the valence model, one detail does not quite conform—turnover. VSGs are very stable and we expected that GPI^2^ E6^R^ and E6^R^:E7^G^ would be equally long lived. However, while both are twice as stable as GPI^1^ E6^R^:E7^R^ and GPI^0^ E7^R^ hetero/homodimers (*t*_1/12_ ~4 hr vs ~2 hr), neither is nearly as stable as VSG. Thus, while our work indicates that there is nothing special about VSG in terms of accessing the cell surface, other than two GPI anchors, there is something unique in terms of avoiding degradation. This may be related to the additional membrane proximal C-terminal domain in VSG that is absent in TfR. This domain, which is less variable than the larger N-terminal domain, might have conserved features that enhance stability, either by favoring recycling to the cell surface or by resisting sorting to the lysosome. Whatever the explanation, the higher turnover rate likely explains the failure of our several attempts to replace the resident VSG221 gene in the BES1 expression site with homodimeric E6.

The finding that E6^R^:E7^G^ forms functional TfR heterodimers is remarkable. Direct binding requires de novo receptor synthesis in the absence of Tf ligand, indicating that all pre-existing cell surface TfR must be saturated during in vivo growth in 10–20% serum. Binding to E6^R^:E7^G^ in the context of the densely packed VSG surface coat is consistent with mutagenesis and molecular modeling studies that place the Tf ligand binding site distal to the plasma membrane [37, 38]. Furthermore, because TfR is smaller than VSG, the structural model dictates that Tf must gain access to a ligand-binding site that is recessed within the surrounding surface coat. Our results confirm this as a realistic model. TfR is thought to be evolutionarily derived from VSG [39, 40], and the fact that it is functional with two GPI anchors begs the question of what selection pressure drove the truncation of ESAG7 and the loss of one anchor, or conversely why VSG is a GPI^2^ homodimer? The answer to the later question is clearly that dimerization is necessary because a single dimyristoyl GPI anchor is unstable in the plasma membrane of BSF trypanosomes. The simplest answer to the first is that in the face of the host adaptive immune response it would be detrimental to have an invariant antigen on the cell surface. Having one GPI anchor assures that any TfR that might exit the flagellar pocket onto the cell surface will be rapidly shed. This may be true, but trypanosomes have other trans-membrane invariant antigens that cannot be shed and that are modeled to protrude from the surrounding VSG coat [40]. Clearly there are other as yet undetermined factors that contribute to immune evasion by trypanosomes.

We have previously proposed a simple mechanism for GPI-dependent post-Golgi trafficking [for a more extensive treatment see [16]]. GPI^0^ cargo trafficks by default to the lysosome as we have seen for many reporters lacking specific retention or targeting signals [16, 19, 32, 34, 41]. GPI^2^ cargo, of which VSG is the exemplar, trafficks rapidly to the flagellar pocket and then diffuses laterally out to the cell surface. Clearly this is facilitated by valence since native TfR (GPI^1^) does not exit the flagellar pocket, while the nearly identical E6^R^:E7^G^ (GPI^2^) does. GPI^1^ cargoes, which parse between the lysosome and the cell surface, are free to dissociate from internal membranes at any time during intracellular transport, but are just as likely to re-associate. This is also true once they reach the flagellar pocket, as indicated by EM studies that consistently show both membrane-bound and lumenal pools of TfR [13, 15, 16, 42]. In either state, GPI^1^ reporters can then be endocytosed and we propose that if membrane associated in endosomal compartments they are likely to be recycled back to the pocket. If they are dissociated from the membrane they will eventually reach a point in endosomal trafficking where subsequent delivery to the lysosome is committed, much as for other soluble fluid phase cargo. Alternatively, GPI^1^ cargo can exit the flagellar pocket by lateral diffusion, at which point dissociation will be essentially irreversible. Exit from the pocket in the soluble state is less likely as we have consistently found that secretion of bona fide soluble secretory reporters is severely constrained in bloodstream trypanosomes, presumably due to pocket architecture [16]. We propose that it is the physical properties of each molecule that ultimately determines the fate of any given GPI^1^ reporter. For instance, native TfR is a large and highly glycosylated dimeric protein that is unable to exit the pocket. Conversely, BiPN:GPI is a small globular non-glycosylated reporter that is mostly shed into the media (~80%). It should be noted that the secretion rate of soluble BiPN is ~50%, confirming that even one GPI anchor can enhance exit from the pocket if the correct reporter is used. This is perhaps a simplified model, but it does account for all our observations. However, whatever the mechanism for post-Golgi sorting of GPI anchored cargo in bloodstream trypanosomes, it is unlikely to be mediated by sterol/sphingolipid-rich rafts, as in polarized epithelial cells [5], since VSG does not enter into Triton X100 insoluble complexes [43], nor does inhibition of sphingolipid synthesis impact its normal transport [44, 45].

Finally, how well do other endogenous GPI anchored proteins conform to the valence model for post-Golgi sorting? One such protein is the haptoglobin-hemoglobin receptor (HpHbR), an essential nutrient receptor for heme acquisition, and the portal of entry for the innate primate immune factor, trypanolytic factor [40, 46, 47]. HpHbR is a monomeric GPI^1^ protein that localizes predominantly to the flagellar pocket. Nothing is known about its turnover, but based on localization alone it apparently fits our model. Another is the serum resistance associated protein, SRA, which confers resistance to trypanolytic factor in human infective trypanosome species. Like TfR, SRA is VSG related and localizes to endosomal compartments, but unlike TfR is modeled to be a homodimer [48, 49]. However, its quaternary structure has never been empirically confirmed, thus its GPI valence is uncertain. One might predict based on localization alone that SRA will be either a GPI^1^ monomer or heterodimer, but further investigation will be required to determine if it fits the model or is an exception. And undoubtedly other GPI anchored proteins will be discovered and characterized in trypanosomes. A cautious scientist would assume that these will not all adhere strictly to the model, but we are confident that our work with VSG, TfR and other engineered GPI reporters lays a general foundation for understanding post-Golgi trafficking of GPI anchored proteins in bloodstream form trypanosomes.

## Materials and methods

### Maintenance and manipulation of trypanosomes

All experiments were carried out with the tetracycline-responsive single-marker (SM) derivative of bloodstream form Lister 427 strain *T*. *brucei brucei* (MITat1.2 expressing VSG221) [50], grown at 37°C in HMI9 medium [51]. For experiments, cells were harvested at mid-late log phase (0.5x10^6^ to 10^6^). Generation of the TfR RNAi cell line using SM cells as the parental cell line has been described in [28]. Cells were grown under antibiotic selection as appropriate. Induction of anti-TfR double-stranded RNA was achieved by addition of 1 μg/ml of tetracycline.

### Construction of RNAi-resistant TfR subunits

All constructs are schematically represented in Fig 1B & 1C. RNAi resistant (RNAi^R^) ESAG7 (E7^R^), ESAG6 (E6^R^) or ESAG7-GPI (E7^G^ –fusion of E6 C-terminus to C-terminus of E7) constructs were cloned into our pXS6 vector [32]. All TfR segments were PCR amplified from H25N7 BAC DNA containing the BES1 expression site [clone H25N7, [52], gift of Gloria Rudenko] as template. Briefly, the E7 genomic replacement construct was assembled as follows (5’-3’): 5’ upstream targeting regions (nts -489 to 1; relative to the E7 ORF); puromycin resistance cassette; βα-tubulin intergenic region; the E7 ORF including the native signal sequences (nts 1–1023, codons 1–341); 3’ downstream targeting region (nts 1–524; relative to E7 stop codon). All segments were confirmed by sequencing. To recode the E7 reporter for RNAi resistance the N-terminal region from the start codon (SnaBI) to an internal BamHI site (nt 739) was chemically synthesized (Integrated DNA Technologies, Coralville, IO), synonymously altering all codons to the next most frequently used codon in *T*. *brucei* housekeeping genes [53, 54]. This synthetic DNA was placed in the E7 construct using SnaBI/BamHI and is referred to hereafter as E7^R^. The E6 genomic replacement construct was created as described above for E7 except for the following: 5’ upstream targeting regions (nts -484 to 1; relative to the E6 ORF); hygromycin resistance cassette; βα-tubulin intergenic region; the E6 ORF including the native signal sequences (nts 1–1206), codons 1–402); 3’ downstream targeting region (nts 1–601; relative to E6 stop codon). The synthetic recoded E6 reporter from start codon (SnaBI) to the internal BamH1 site (nt 742) was cloned (SnaBI/BamHI) into the E6 construct to generate E6^R^. To alter the GPI status of E7, the E6^R^ construct was digested with SacI/MfeI (internal SacI to the stop codon) and cloned into the corresponding E7^R^ construct with the same restriction enzymes creating E7-GPI (denoted as E7^G^). Alignment of wild type and RNAi^R^ full length sequences are presented in S1 Fig. The resultant RNAi^R^ reporters (E6^R^, E7^R^, and E7^G^) were linearized with ClaI/FseI for homologous replacement of the endogenous respective genes in the active ES1 expression site of the TfR RNAi cell line (Fig 1B) [28]. Transfection and clonal selection with appropriate antibiotics was performed was described in [28].

### Immunological reagents

The following antibodies have been described in our prior publications [28, 32]: rabbit anti-VSG221, mouse anti-BiP, and anti-HSP70. Rabbit anti-TfR (ES1 specific) was a generous gift of Dr. Piet Borst (Netherlands Cancer Institute, Amsterdam). Secondary reagents for western blotting were IRDye680- and IRDye800-conjugated goat anti-rabbit and anti-mouse IgG (*Li-Cor*, Lincoln NB). Secondary reagents for immunofluorescent imaging were species-specific Alexa-conjugated goat anti-IgG as appropriate (Molecular Probes, Eugene, OR).

### Metabolic labeling and pull-downs

Pulse-chase radiolabeling of log-phase cultured BSF trypanosomes with [^35^S]methionine/cysteine; Perkin Elmer, Waltham, MA], and subsequent immunoprecipitation of labeled polypeptides were performed as described previously [55, 56]. As indicated, cells were pre-treated (15 min) and radiolabeled as described above in the continued presence of the thiol protease inhibitor FMK024 (morpholinourea-phenylalanine-homophenylalanine-fluoromethyl ketone; 20 μM; MP Biomedicals, Aurora, OH). Pulse and chase times are indicated in the figure legends. For TfR dsRNA and RNAi^R^ subunit expression, control and tetracycline-induced cells (0 or 24 hrs) were radiolabeled for 1 hr. Radiolabeled TfR (native or RNAi^R^) polypeptides were subjected to pull-downs with transferrin-conjugated beads (Tf-beads), anti-TfR or anti-HSP70. All pull-downs were fractionated by 12% SDS-PAGE, and gels were analyzed by phosphorimaging using a Molecular Dynamics Typhoon FLA 9000 system with native ImageQuant Software (GE Healthcare, Piscataway, NJ).

### Endocytosis assay

Endocytosis was assayed by flow cytometry as generally described in [32]. Washed log-phase cells (10^6^/ml) were pre-incubated (10 min, 37°C) in serum free HMI9 medium with 0.5 mg/ml BSA. Ligands (Alexa488 conjugated bovine transferrin or tomato lectin, 5 μg/ml, Molecular Probes) were added and incubation was continued for 30 minutes. Cells were then processed for flow cytometry.

### Immunoblotting

Gels were transferred to Immobilon-P membranes (Millipore Corp., Bedford, MA) using a Trans-Blot Turbo apparatus (BioRad, Hercules, California). Membranes were blocked and probed with appropriate dilutions of primary and secondary antibodies in Odyssey Blocking Buffer (*Li-Cor*). All washes were with PBS, 0.5% Tween20. Quantitative fluorescent signals were scanned on an Odyssey CLx Imager (*Li-Cor*).

### qRT-PCR

Specific transcript levels were determined using quantitative RT-PCR (qPCR). Total RNA was isolated from log phase cultures using RNeasy Mini kit (Qiagen, Valencia, CA). RNA was treated with DNAse1 on-column using RNase-Free DNase Set (Qiagen) and cDNA synthesized using iScript cDNA synthesis kit (BioRad, Hercules, CA). qPCR was performed using diluted cDNAs and Power SYBR green PCR Master Mix (Life Technologies, Carlsbad, CA) with oligonucleotide pairs specifically targeting transcripts for native E6^N^ and E7^N^, and RNAi^R^ E6^R^ and E7^R^. The positions of these primers are indicated in the sequence alignment presented in S1 Fig. TbZFP3 (Tb927.3.720, nts 241–301) was used as the control amplicon. Amplification was performed using an Applied Biosystems StepOne Real-Time PCR System (Life Technologies, Carlsbad, CA). For each transcript post-amplification melting curves indicated a single dominant product. All calculations and normalizations were done using StepOne software, version 2.2.2. Reactions were performed in technical triplicates, and means ± standard errors of the means (SEM) for three biological replicates are presented.

### Epifluorescence microscopy

Immunofluorescence (IFA) microscopy was performed with formaldehyde fixed/detergent permeablized cells as described in [55]. Cells were also stained with DAPI (0.5 μg ml^-1^) to reveal nuclei and kinetoplasts. Serial image stacks (0.2 micron Z-increment) were collected with capture times from 100–500 msec (100x PlanApo, oil immersion, 1.46 na) on a motorized Zeiss Axioimager M2 stand equipped with a rear-mounted excitation filter wheel, a triple pass (DAPI/FITC/Texas Red) emission cube, differential interference contrast (DIC) optics, and an Orca ER CCD camera (Hamamatsu, Bridgewater, NJ). Images were collected with Volocity 6.1 Acquisition Module (Improvision Inc., Lexington, MA) and individual channel stacks were deconvolved by a constrained iterative algorithm, pseudocolored, and merged using Volocity 6.1 Restoration Module. Unless otherwise stated all images presented are summed stack projections of merged channels. The xyz pixel precision of this arrangement has been validated in [18] (see S1 Fig therein).

### Transferrin binding

Cells grown in HMI9 medium were harvested, washed with HEPES buffered-saline (HBS) supplemented with 1% w/v glucose [57], and incubated at 37°C (2 hr, 5x10^6^ cells/ml) in HMI9/BSA. This treatment was necessary to replace existing ligated surface TfR with newly synthesized unligated TfR. During the pre-incubation period, the cells were untreated (control), or treated with cycloheximide (CHX, 100 μg/ml) to block protein synthesis. For flow cytometry and epifluorescence microscopy cells were then washed with ice cold PBS with 1% w/v glucose (PBSG) and incubated with Tf488 (Molecular Probes, 2 μg/ml, 1 hr, 4°C) without or with 100x excess holotransferrin as competitor. Cells were then processed for either flow cytometry or microscopy as described above. For scanning electron microscopy, Tf-colloidal gold (Cytodiagnostics, Burlington, ON, Canada, 100 nm) was concentrated by centrifugation and added directly to cells (final 3.75 mg/ml, 7.5 OD) following the preincubation step. Incubation was continued an additional 1 hr at 4°C and cells were processed directly for electron microscopy as described below.

### Scanning electron microscopy (SEM)

For electron microscopy, cells were stained with Tf:gold as described above. The fixation/dehydration protocol is described in [58] with the following modifications. Cells were fixed in HMI9/BSA (2 hrs, 4°C) in 2.5% EM grade glutaraldehyde. Post fixation, cells were collected by syringe-passage onto 0.2 μm pore polycarbonate filters (Whatman Nucleopore, 25 mm dia., SIGMA-ALDRICH, St. Louis, MO) keeping fluid in the upper filter chamber (Whatman Swin-Lok Cartridge, 25 mm, SIGMA-ALDRICH) in all subsequent steps until final air drying. Washing and fixation were done through the filter as follows: 5 ml 2.5% glutaraldehyde in PBS allowing rest of 10 mins; 10 ml PBS rest 10 mins; 5 ml 30% v/v, 50% v/v, 70% v/v, 90% v/v ethanol in water 5 mins each; 5 ml 100% ethanol twice 5 mins each. Samples were then dried with hexamethyldisilazane (HMDS, 5 mls, 5 mins). Filters were removed, air dried, and coated with evaporated carbon at high vacuum (Denton 502 evaporator). Cells were imaged with a Hitachi SU70 FESEM at 20 KeV using combined signals from a conventional Everhart-Thornley detector (adjusted to maximize backscattered electron component) and in-lens secondary electron detector. The combined signal showed gold nanoparticles as bright dots superimposed on cell surface morphology.

### Blue native polyacrylamide gel electrophoresis (BN-PAGE)

BN-PAGE was performed using the NativePAGE Bis-Tris Gel System (Thermo Fisher Scientific, Waltham, MA). Briefly, cells were harvested, washed with HBS and solubilized in NativePAGE Sample Buffer supplemented with 10% glycerol, 1% DDM (n-dodecyl-β-D-maltoside), 1X protease inhibitor cocktail and 100 μg/ml DNaseI. The samples were incubated in the solubilization buffer on ice for 30 min, centrifuged (13000g at 4°C, 1 hr), and the resulting supernatants were either untreated or treated with 4M urea to denature protein complexes. Samples were then fractionated on precast 4–16% BN gradient gels (Thermo Fischer Scientific). After electrophoresis, proteins were transferred to PVDF membranes (Millipore Corp., Bedford, MA) and detected by our standard immunoblotting protocol with anti-TfR or anti-VSG221.

### Data analyses

Phosphorimages and fluorescent blot scans were quantified with ImageJ software (http://imagej.nih.gov/ij/). For analysis of specific band intensities, signals were corrected by subtraction of the signal from equivalent unlabeled areas of each lane. All subsequent data management was performed with Prism4 software (GraphPad Software, Inc., San Diego CA).

## Supporting information

S1 Fig Alignment of E6^N^, E7^N^, E6^R^ and E7^R^ sequences. The native E6^N^ and E7^N^ sequences (black) are aligned to each other from the native N-terminal signal sequence cleavage sites to the C-terminal stop codons, and to recoded RNAi^R^ resistant E6^R^ and E7^R^ from the native signal sequence to the internal BamHI site (underlined). The positions of specific forward (grey shading) and reverse (yellow shading) used for qRT-PCR of each ORF are indicated. Dashes indicate gaps in the alignment. Dots indicate identity with native E6 or E7. Native TfR sequences are derived from the BES1 telomere [52].
(PDF)

S2 Fig Dimerization of RNAi^R^ TfR subunits. RNAi resistant cell lines as indicated were cultured with tetracycline for 24 hr, extracted with 1% dodecylmaltoside, incubated without (-) or with 4 M urea (+), and fractionated by BN-PAGE. Gels were transferred to membranes and immunoblotted with anti-TfR (**A**) or anti-VSG221 (**B**). Each lane contains 10^6^ cell equivalents. Mobilities of TfR dimers (dTfR), TfR monomers (mTfR: E6^R^, E7^G^, E7^R^), dimeric VSG (dVSG), and monomeric VSG (mVSG) are indicated on the left. Mobilities of molecular mass markers are indicated on the right. All matched (TfR vs. VSG) urea +/- lanes are from the same blots and images. White lines indicate lanes that were digitally excised after image processing in order to clarify presentation. Representative images are presented.
Endogenous VSG221 serves as an internal control, and in each case is detected as a dimer of appropriate mass (~120 kDa) that dissociates to monomers (~60 kDa) with urea treatment. Small amounts of monomeric VSG are present in each native extract. Whether this represents the in vivo condition or dissociation due to experimental handling is not clear. As expected, TfR from E6^R^:E7^R^ cells appears quantitatively as a heterodimer of appropriate mass (smaller than dVSG), and dissociates to component subunits of expected masses (E6^R^ > E7^R^). E7^R^ TfR presents a more complex profile, primarily as a species smaller than native TfR, consistent with homodimerization, with a small amount of free monomer. However, a smear of higher mass material is present in the non-denatured sample. Urea treatment generates more E7^R^ monomer, but resistant dimers and smear remains. This is highly reproducible. We conclude that folding/dimerization of E7^R^ is less efficient when expressed discretely, and consequently that significant misfolding/aggregation results. E6^R^ TfR is predominantly a single species with mobility intermediate to that of VSG and normal TfR. It dissociates to a single E6^R^ species, consistent with the formation of homodimers. Finally, TfR from the E6^R^:E7^G^ cell line presents as a single heterodimeric species of mass similar to E6^R^ homodimers. Urea treatment generates an apparent single species containing both the E6^R^ and E7^G^ subunits, which are essentially the same size (see Fig 1C). Overall these results confirm the expected hetero- and homodimeric states of the various TfR constructs. It is notable that each TfR species, including the normal E6^R^:E7^R^ heterodimer, is considerably more resistant to denaturation that native VSG, suggesting stronger internal physical interactions.
(TIF)

S3 Fig Flow cytometry of TfR surface expression. The E6^R^ (**A**) and E6^R^:E7^G^ (**B**) cell lines were silenced for 24 hrs, stained with specific primary antibodies as indicated and then analyzed by flow cytometry with A488-conjugated goat anti-rabbit IgG. Each histogram represents 50,000 events. Red, no primary control; blue, anti-TfR; green, anti-VSG221. Solid lines, tet+; dashed lines, tet-. Analyses for each cell line were on separate days and cannot be directly compared.
(TIF)

S4 Fig TfR GPI anchors. Bloodstream form trypanosomes have an endogenous GPI-specific phospholipase C (GPI-PLC) activity, which is tightly regulated in intact cells, and which has been studied in regard to membrane association of the major GPI-anchored protein, variant surface glycoprotein (VSG) [59, 60]. GPI hydrolysis removes dimyristoylglycerol, leaving behind a 1’, 2’ cyclic inositol monophosphate [17], and converting native VSG from membrane-form (mfVSG) to soluble-form (sVSG) [61] The residual GPI structure on sVSG, but not the intact structure on mfVSG, forms a cross-reacting determinant (CRD) that reacts with specific anti-CRD antibodies present in hyperimmune sera of rabbits immunized with sVSG [62]. Such reactivity is diagnostic for the presence of a GPI anchor, and cell lysates can be prepared in which GPI anchors are all hydrolysed (s-lysis, CRD+) or all intact (mf-lysis, CRD-) [20, 63]. These properties hold for any GPI-anchored protein in BSF trypanosomes, and form the basis for our analyses of the GPI status of our TfR reporters.
TfR cell lines were lysed as follows: For s-lysis washed cells were suspended at 1x10^8^ cells/ml in TEN buffer (50 mM TrisHCl, pH 7.5, 150 mM NaCl, 5 mM EDTA) containing 1% NP40 and protease inhibitor cocktail (PIC). Lysates were incubated at 37°C for 5 minutes to allow complete hydrolysis of all GPI anchors, and were then adjusted to final detergent conditions for immunoprecipitation (1x10^7^ cells/ml in TEN containing 1% NP40, 0.5% deoxycholate, 0.1% SDS, PIC). For mf-lysis cells were suspended at 1x10^8^ cells/ml in TEN containing 1% SDS with PIC and boiled for 5 minutes to denature endogenous GPI-PLC. Lysates were cooled and adjusted to final detergent conditions as defined above. All lysates were clarified by centrifugation prior to immunoprecipitation.
**A**. Lysates (s and mf as indicated) from E6^R^:E7^R^, E7^R^, E6^R^ and E6^R^:E7^G^ RNAi^R^ cell lines (as indicated) were immunoprecipitated with anti-VSG221 antibodies covalently cross-linked to protein A sepharose (5x10^5^ cell equivalents/precipitate). Precipitates were prepared for immunoblotting by standard SDS-PAGE, trans-blotting, and blocking. Membranes were first probed with anti-VSG (top) and then stripped and reprobed with anti-CRD antibody [bottom, rabbit anti-AnTat1.8 sVSG affinity purified on ILTat1.1 sVSG sepharose [63]. Blots were imaged by chemiluminescence. Mobilities of VSG (V) and molecular mass markers (kDa) are indicated. Equal amounts of VSG were detected in all lysates (top) indicating equal recovery and loading for all matched s- and mf-lysates. However, only VSG prepared by s-lysis was reactive with anti-CRD (A, bottom) confirming the validity of our protocol.
**B**. Duplicate aliquots of s-lysates from silenced E6^R^:E7^R^, E6^R^, E7^R^ and E6^R^:E7^G^ cell lines were immunoprecipitated with anti-TfR antibodies covalently cross-linked to protein A sepharose (1x10^7^ cell equivalents/precipitate). One set of precipitates was immunoblotted with anti-TfR, and the other set was immunoblotted with anti-CRD. TfR polypeptides of the appropriate relative sizes were detected in all cases indicating equal recovery and loading (left). The weak E6 signal in the E7^R^ cell line likely represents residual native E6. Anti-CRD reactivity was detected for E6^R^ and/or E7^G^ in the E6^R^:E7^R^, E6^R^, and E6^R^:E7^G^ cell lines, but not the E7^R^ cell line (right). The lack of reactivity of non-GPI-anchored E7 polypeptides confirms the specific of the anti-CRD reagent. The weak E6 signal likely represents residual native E6 following RNAi silencing. These results confirm proper GPI attachment for all E6^R^ and E7^G^ polypeptides as expected for each cell line. Mobilities of TfR subunits and molecular mass markers (kDa) are indicated.
(TIF)
