# Supplementary material for: Controlling transferrin receptor trafficking with GPI-valence in bloodstream stage African trypanosomes
Source: PLoS Pathog. 2017 May 1;13(5):e1006366. doi: 10.1371/journal.ppat.1006366 (PMC5426795; doi:10.1371/journal.ppat.1006366)
Supplement: S1 Fig — The native E6N and E7N sequences (black) are aligned to each other from the native N-terminal signal sequence cleavage sites to the C-terminal stop codons, and to recoded RNAiR resistant E6R and E7R from the native signal sequence to the internal BamHI site (underlined). The positions of specific forward (grey shading) and reverse (yellow shading) used for qRT-PCR of each ORF are indicated. Dashes indicate gaps in the alignment. Dots indicate identity with native E6 or E7. Native TfR sequences are derived from the BES1 telomere [52]. (PDF) [file ppat.1006366.s001.pdf]

S1. E6/E7 WT vs RNAi^R Sequence Alignment

```
        M   M   K   F   W   F   V   L   L   A   L   L   G   K   E   T   H   A      18
E6WT   ATG ATG AAG TTT TGG TTT GTG CTG TTG GCT CTT TTG GGA AAA GAA ACA CAT GCA     54
E6R    ATG ATG AAA TTC TGG TTC GTC CTC CTC GCG CTC CTT GGG AAG GAG ACT CAC GCG
E7WT   ATG ATG AAG TTT TGG TTT GTG CTG TTG GCT CTT TTG GGA AAA GAA ACA CAT GCA
E7R    ATG ATG AAA TTC TGG TTC GTC CTC CTC GCG CTC CTT GGG AAG GAG ACT CAC GCG
        M   M   K   F   W   F   V   L   L   A   L   L   G   K   E   T   H   A

        Y | Y   E   N   K   R   N   A   L   N   A   T   A   A   N   K   V   C      36
E6WT   TAT TAT GAA AAT AAA AGG AAT GCA TTA AAT GCA ACC GCC GCT AAT AAA GTG TGT    108
E6R    TAC TAC GAG AAC AAG CGA AAC GCG CTC AAC GCG ACT GCG GCG AAC AAG GTT TGC
E7WT   TAT TAT GAA AAT AAA AGG AAT GCA TTA AAT GCA ACC GCC GCT AAT AAA GTG TGT
E7R    TAC TAC GAG AAC AAG CGA AAC GCG CTC AAC GCG ACT GCG GCG AAC AAG GTT TGC
        Y | Y   E   N   K   R   N   A   L   N   A   T   A   A   N   K   V   C

        G   L   S   T   Y   L   K   G   I   A   H   R   V   N   S   E   S   A      54
E6WT   GGG CTA TCG ACC TAT CTT AAA GGA ATA GCG CAC AGA GTA AAC AGC GAG AGT GCC    162
E6R    GGA TTG AGC ACT TAC TTG AAG GGG ATC GCA CAT CGG GTC AAT TCA GAA TCA GCT
E7WT   GGG CTA TCG ACC TAT CTT AAA GGA ATA GCG CAC AGA GTA AAC AGC GAG AGT GCC
E7R    GGA TTG AGC ACT TAC TTG AAG GGG ATC GCA CAT CGG GTC AAT TCA GAA TCA GCT
        G   L   S   T   Y   L   K   G   I   A   H   R   V   N   S   E   S   A

        V   V   T   E   K   L   S   D   L   K   M   R   S   I   Q   L   Q   L      72
E6WT   GTG GTT ACG GAA AAA CTA TCA GAT TTG AAA ATG AGA AGC ATC CAG TTG CAG TTA    216
E6R    GTT GTC ACA GAG AAG TTG AGC GAC CTC AAG ATG CGA TCA ATT CAA CTC CAA CTC
E7WT   GTG GTT ACG GAA AAA CTA TCA GAT TTG AAA ATG AGA AGC ATC CAG TTG CAG TTA
E7R    GTT GTC ACA GAG AAG TTG AGC GAC CTC AAG ATG CGA TCA ATT CAA CTC CAA CTC
        V   V   T   E   K   L   S   D   L   K   M   R   S   I   Q   L   Q   L

        S   V   M   R   N   R   V   P   S   G   E   Q   D   C   K   D   I   R      90
E6WT   TCA GTA ATG CGG AAC AGA GTG CCT TCT GGC GAG CAG GAT TGT AAA GAC ATC AGG    270
E6R    AGC GTC ATG AGG AAT CGG GTT CCC AGC GGT GAA CAA GAC TGC AAG GAT ATT CGA
E7WT   TCA GTA ATG CGG AAC AGA GTG CCT TCT GGC GAG CAG GAT TGT AAA GAC ATC AGG
E7R    AGC GTC ATG AGG AAT CGG GTT CCC AGC GGT GAA CAA GAC TGC AAG GAT ATT CGA
        S   V   M   R   N   R   V   P   S   G   E   Q   D   C   K   D   I   R

        T   L   L   K   T   V   L   R   N   E   F   T   F   Q   Q   E   L   E     108
E6WT   ACA CTC TTG AAA ACA GTA TTG AGG AAT GAG TTT ACA TTC CAG CAG GAG TTG GAG    324
E6R    ACG TTG CTC AAG ACC GTC CTC CGA AAC GAA TTC ACC TTT CAA CAA GAA CTC GAA
E7WT   ACA CTC TTG AAA ACA GTA TTG AGG AAT GAG TTT ACA TTC CAG CAG GAG TTG GAG
E7R    ACG TTG CTC AAG ACC GTC CTC CGA AAC GAA TTC ACC TTT CAA CAA GAA CTC GAA
        T   L   L   K   T   V   L   R   N   E   F   T   F   Q   Q   E   L   E

        E   M   R   N   A   S   A   L   A   A   A   A   A   G   I   A   A   G     126
E6WT   GAA ATG AGG AAC GCA TCC GCG TTA GCA GCA GCT GCA GCT GGG ATA GCA GCT GGA    378
E6R    GAG ATG CGA AAT GCG AGT GCA CTC GCG GCT GCC GCG GCC GGA ATC GCG GCC GGG
E7WT   GAA ATG AGG AAC GCA TCC GCG TTA GCA GCA GCT GCA GCT GGG ATA GCA GCT GGA
E7R    GAG ATG CGA AAT GCG AGT GCA CTC GCG GCT GCC GCG GCC GGA ATC GCG GCC GGG
        E   M   R   N   A   S   A   L   A   A   A   A   A   G   I   A   A   G

        R   L   E   E   W   I   F   V   F   A   Q   A   A   G   G   S   S   Q     144
E6WT   AGA CTG GAA GAG TGG ATT TTT GTA TTT GCA CAG GCA GCA GGC GGG TCA TCA CAG    432
E6R    CGG CTC GAA GAA TGG ATC TTC GTC TTC GCG CAA GCG GCG GGT GGA AGC AGC CAA
E7WT   AGA CTG GAA GAG TGG ATT TTT GTA TTT GCA CAG GCA GCA GGC GGG TCA TCA CAG
E7R    CGG CTC GAA GAA TGG ATC TTC GTC TTC GCG CAA GCG GCG GGT GGA AGC AGC CAA
        R   L   E   E   W   I   F   V   F   A   Q   A   A   G   G   S   S   Q

        F   C   I   S   V   G   T   N   I   P   A   E   Y   N   N   L   Q   E     162
E6WT   TTT TGC ATA AGC GTA GGG ACG AAT ATC CCA GCA GAA TAC AAC AAC TTG CAA GAA    486
E6R    TTC TGT ATC TCA GTC GGA ACA AAC ATT CCG GCG GAG TAT AAT AAT CTC CAG GAG
E7WT   TTT TGC ATA AGC GTA GGG ACG AAT ATC CCA GCA GAA TAC AAC AAC TTG CAA GAA
E7R    TTC TGT ATC TCA GTC GGA ACA AAC ATT CCG GCG GAG TAT AAT AAT CTC CAG GAG
        F   C   I   S   V   G   T   N   I   P   A   E   Y   N   N   L   Q   E
```

```
      C   F   D   G   I   I   G   P   E   T   L   Y   K   I   E   D   S   R     180
E6WT  TGT TTT GAT GGA ATA ATT GGA CCT GAA ACC CTT TAC AAA ATT GAG GAT TCA CGT   540
E6R   TGC TTC GAC GGC ATC ATC GGA CCC GAG ACT TTG TAT AAG ATC GAA GAC AGC CGC
E7WT  TGT TTT GAT GGA ATA ATT GGA CCT GAA ACC CTT TAC AAA ATT GAG GAT TCA CGT
E7R   TGC TTC GAC GGC ATC ATC GGA CCC GAG ACT TTG TAT AAG ATC GAA GAC AGC CGC
      C   F   D   G   I   I   G   P   E   T   L   Y   K   I   E   D   S   R

      V   K   E   S   A   Q   K   S   L   Q   L   H   E   V   L   S   S   I     198
E6WT  GTA AAA GAG TCG GCG CAG AAA AGC TTG CAA CTC CAT GAA GTG TTA TCA TCC ATT   594
E6R   GTC AAG GAA AGC GCA CAA AAG TCA CTC CAG TTG CAC GAG GTT CTC AGC AGT ATC
E7WT  GTA AAA GAG TCG GCG CAG AAA AGC TTG CAA CTC CAT GAA GTG TTA TCA TCC ATT
E7R   GTC AAG GAA AGC GCA CAA AAG TCA CTC CAG TTG CAC GAG GTT CTC AGC AGT ATC
      V   K   E   S   A   Q   K   S   L   Q   L   H   E   V   L   S   S   I

      S   F   N   S   L   G   A   E   N   I   R   G   G   N   G   R   D   G     216
E6WT  TCC TTC AAT AGC TTG GGT GCG GAA AAT ATC CGA GGA GGA AAT GGG AGG GAT GGA   648
E6R   AGT TTT AAC TCA CTC GGC GCA GAG AAC ATT AGG GGC GGC AAC GGA CGA GAC GGC
E7WT  TCC TTC AGT AGC TTG GGT GCG GAA AGT ATT GTT GAG CAA CGA AAG AAC AGA GGA
E7R   AGT TTT TCA TCA CTC GGC GCA GAG TCA ATC GTG GAA CAG AGG AAA AAT CGG GGC
      S   F   S   S   L   G   A   E   S   I   V   E   Q   R   K   N   R   G

      C   N   L   V   R   T   D   T   D   G   V   L   E   G   G   S   V   R     234
E6WT  TGT AAT TTG GTG AGA ACA GAT ACT GAC GGT GTA TTG GAA GGG GGG TCG GTG AGA   702
E6R   TGC AAC CTC GTC AGG ACA GAC ACC GAT GGC GTC CTC GAA GGA GGA AGC GTC CGG
E7WT  TGT AAC CTA ATG CGG ACG GCT GAC GGA GGT TTG CTG AAA GAT ATT TGT TTG AAT
E7R   TGC AAT TTG ATG AGG ACA GCC GAT GGC GGC CTC CTC AAG GAC ATC TGC CTC AAC
      C   N   L   M   R   T   A   D   G   G   L   L   K   D   I   C   L   N

      R   H   N   L   T   W   G   G   G   V   M   N   F   G   S   Y   Q   N     252
E6WT  CGG CAC AAC TTA ACG TGG GGT GGT GGA GTG ATG AAT TTT GGA TCC TAT CAA AAC   756
E6R   AGG CAT AAT CTC ACG TGG GGC GGC GGC GTT ATG AAC TTT GGA TCC ... ... ...
E7WT  TGC --- AAC TTC ACA TGG GGT GGT GGA GTG ATG AAT TTC GGA TCC TGT GTG GCG
E7R   TGT --- AAT TTT ACG TGG GGC GGC GGC GTT ATG AAC TTT GGA TCC ... ... ...
      C   -   N   F   T   W   G   G   G   V   M   N   F   G   S   C   V   A

      G   S   M   Y   V   E   G   G   E   Y   G   D   A   T   E   Y   G   A     270
E6WT  GGA AGT ATG TAC GTT GAG GGT GGT GAA TAC GGT GAT GCT ACA GAG TAC GGT GCA   810
E7WT  GGG AAC CTA AAA ATA AAA GGG GGA GAA TAC GGT GAT GTC AGT TCT CAC GAT GTG
      G   N   L   K   I   K   G   G   E   Y   G   D   V   S   S   H   D   V

      V   R   W   T   E   D   P   S   K   V   S   I   F   K   D   V   I   R     288
E6WT  GTA CGG TGG ACC GAA GAT CCC AGT AAA GTG AGC ATA TTT AAG GAT GTC ATC CGC   864
E7WT  GTA CGG TGG ACC GAA GAT CCC AGT AAA GTG AGC ATA TTT AAG GAT GTC ATC CGC
      V   R   W   T   E   D   P   S   K   V   S   I   F   K   D   V   I   R

      L   F   A   R   F   Q   E   A   K   N   E   V   M   N   K   I   K   T     306
E6WT  CTG TTT GCG CGC TTC CAA GAA GCA AAA AAT GAA GTG ATG AAT AAA ATA AAA ACT   918
E7WT  CTG TTT GCG CGC TTC CAA GAA GCA AAA AAT GCA GTG ATG AAT AAA ATA AAA ACT
      L   F   A   R   F   Q   E   A   K   N   A   V   M   N   K   I   K   T

      T   V   D   E   L   A   K   C   I   G   Q   K   E   V   E   L   T   D     324
E6WT  ACT GTG GAT GAA TTG GCG AAA TGC ATA GGG CAG AAA GAG GTT GAG CTC ACT GAT   972
E7WT  ACT GTG GAT GAA TTG GCG AAA TGC ATA GGG CAG AAA GAG GTT GAG CTC ACT AAT
      T   V   D   E   L   A   K   C   I   G   Q   K   E   V   E   L   T   N

      D   Q   L   Y   E   E   F   I   W   E   T   I   H   R   L   E   L   S     342
E6WT  GAT CAG CTT TAC GAG GAA TTT ATC TGG GAG ACC ATA CAC AGA TTG GAG CTG TCA  1026
E7WT  GAT CAG CTT TAC GAA GAA TTC GAG GCA ATA CAA AAG TAC CTA GGG TCT CTG tga
      D   Q   L   Y   E   E   F   E   A   I   Q   K   Y   L   G   S   L

      K   R   V   S   E   Q   L   S   L   G   E   E   E   E   T   I   L   K     360
E6WT  AAG AGA GTG AGT GAA CAA CTC TCT TTA GGT GAA GAA GAG GAG ACG ATC CTG AAA  1080
```

```
       S   N   Y   T   A   E   P   V   R   G   P   F   T   V   A   G   S   N   378
E6WT  TCT AAC TAT ACT GCA GAA CCG GTC CGT GGA CCT TTC ACG GTA GCG GGG TCC AAC 1134

       T   V   A   V   H   L   S   L   F   T   A   A   L   C   C   S   A   L   396
E6WT  ACA GTA GCA GTA CAT TTG AGT CTT TTT ACC GCT GCA CTT TGT TGT TCA GCT TTA 1188

       L   L   G   V   L          401
E6WT  TTG TTG GGA GTG CTG tga  1206
```
