# Supplementary figures and images for: Controlling transferrin receptor trafficking with GPI-valence in bloodstream stage African trypanosomes
Source: PLoS Pathog. 2017 May 1;13(5):e1006366. doi: 10.1371/journal.ppat.1006366 (PMC5426795; doi:10.1371/journal.ppat.1006366)

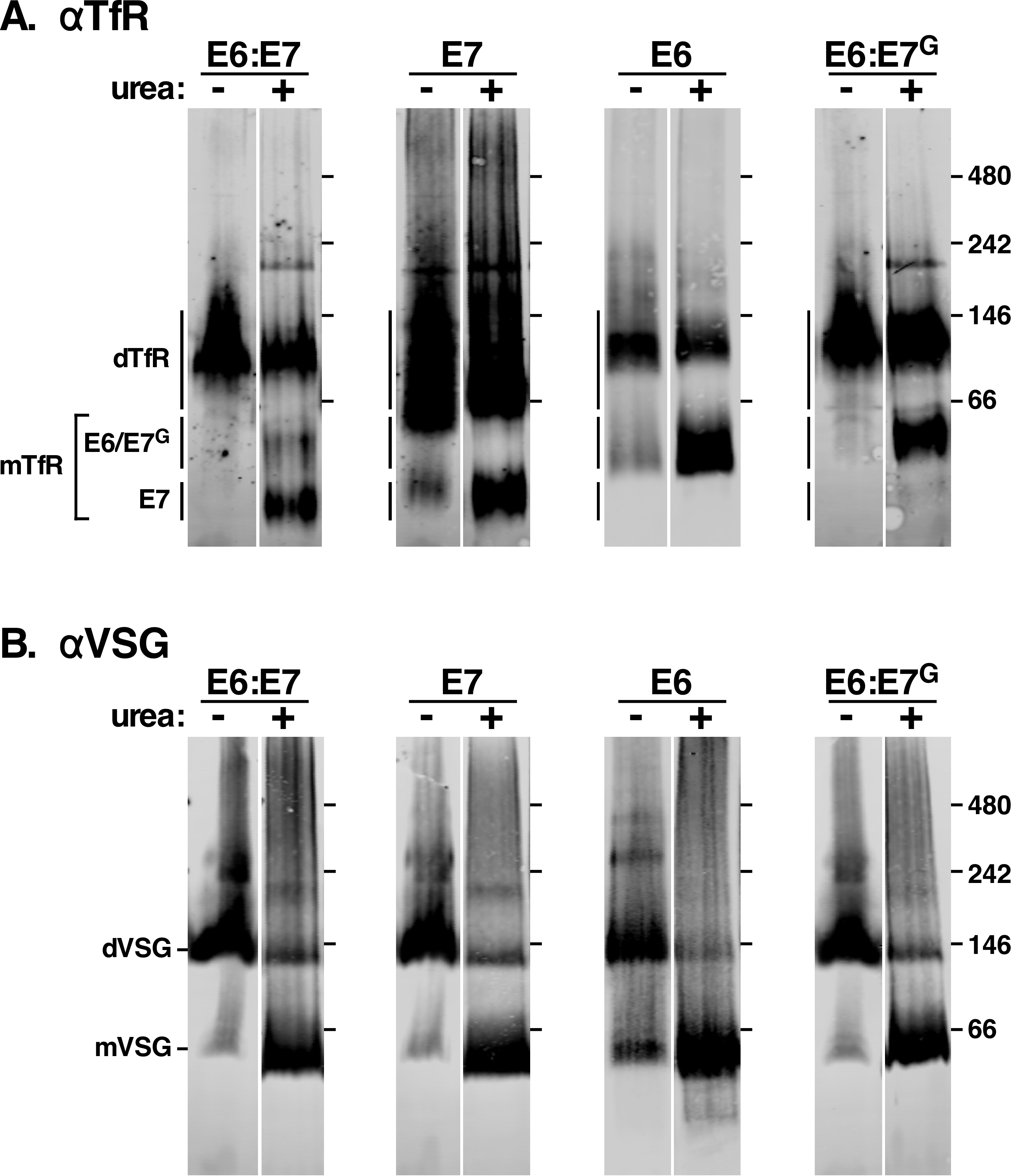

Supplement: S2 Fig — RNAi resistant cell lines as indicated were cultured with tetracycline for 24 hr, extracted with 1% dodecylmaltoside, incubated without (-) or with 4 M urea (+), and fractionated by BN-PAGE. Gels were transferred to membranes and immunoblotted with anti-TfR (A) or anti-VSG221 (B). Each lane contains 106 cell equivalents. Mobilities of TfR dimers (dTfR), TfR monomers (mTfR: E6R, E7G, E7R), dimeric VSG (dVSG), and monomeric VSG (mVSG) are indicated on the left. Mobilities of molecular mass markers are indicated on the right. All matched (TfR vs. VSG) urea +/- lanes are from the same blots and images. White lines indicate lanes that were digitally excised after image processing in order to clarify presentation. Representative images are presented. Endogenous VSG221 serves as an internal control, and in each case is detected as a dimer of appropriate mass (~120 kDa) that dissociates to monomers (~60 kDa) with urea treatment. Small amounts of monomeric VSG are present in each native extract. Whether this represents the in vivo condition or dissociation due to experimental handling is not clear. As expected, TfR from E6R:E7R cells appears quantitatively as a heterodimer of appropriate mass (smaller than dVSG), and dissociates to component subunits of expected masses (E6R > E7R). E7R TfR presents a more complex profile, primarily as a species smaller than native TfR, consistent with homodimerization, with a small amount of free monomer. However, a smear of higher mass material is present in the non-denatured sample. Urea treatment generates more E7R monomer, but resistant dimers and smear remains. This is highly reproducible. We conclude that folding/dimerization of E7R is less efficient when expressed discretely, and consequently that significant misfolding/aggregation results. E6R TfR is predominantly a single species with mobility intermediate to that of VSG and normal TfR. It dissociates to a single E6R species, consistent with the formation of homodimers. Finally, TfR [file ppat.1006366.s002.tif]

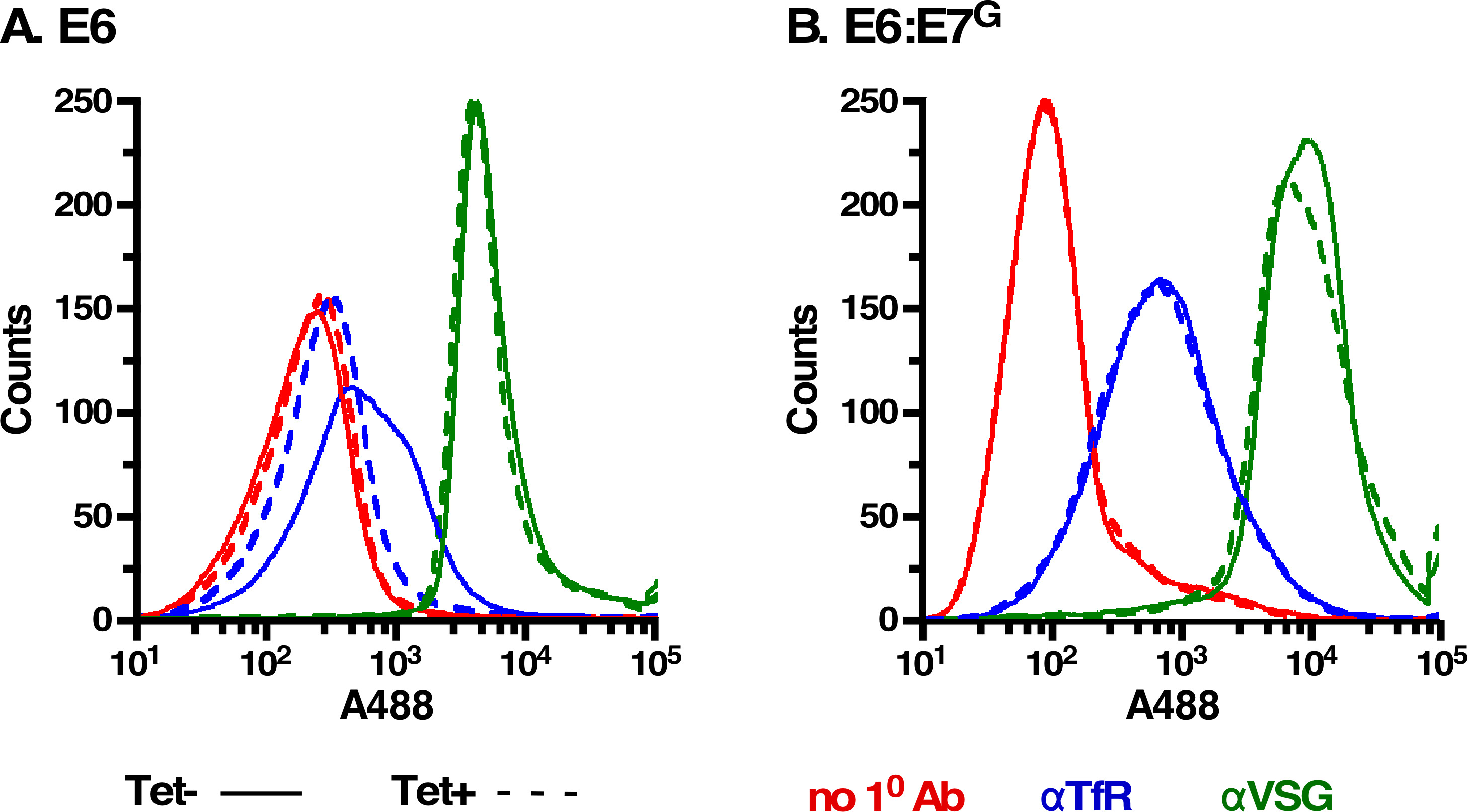

Supplement: S3 Fig — The E6R (A) and E6R:E7G (B) cell lines were silenced for 24 hrs, stained with specific primary antibodies as indicated and then analyzed by flow cytometry with A488-conjugated goat anti-rabbit IgG. Each histogram represents 50,000 events. Red, no primary control; blue, anti-TfR; green, anti-VSG221. Solid lines, tet+; dashed lines, tet-. Analyses for each cell line were on separate days and cannot be directly compared. (TIF) [file ppat.1006366.s003.tif]

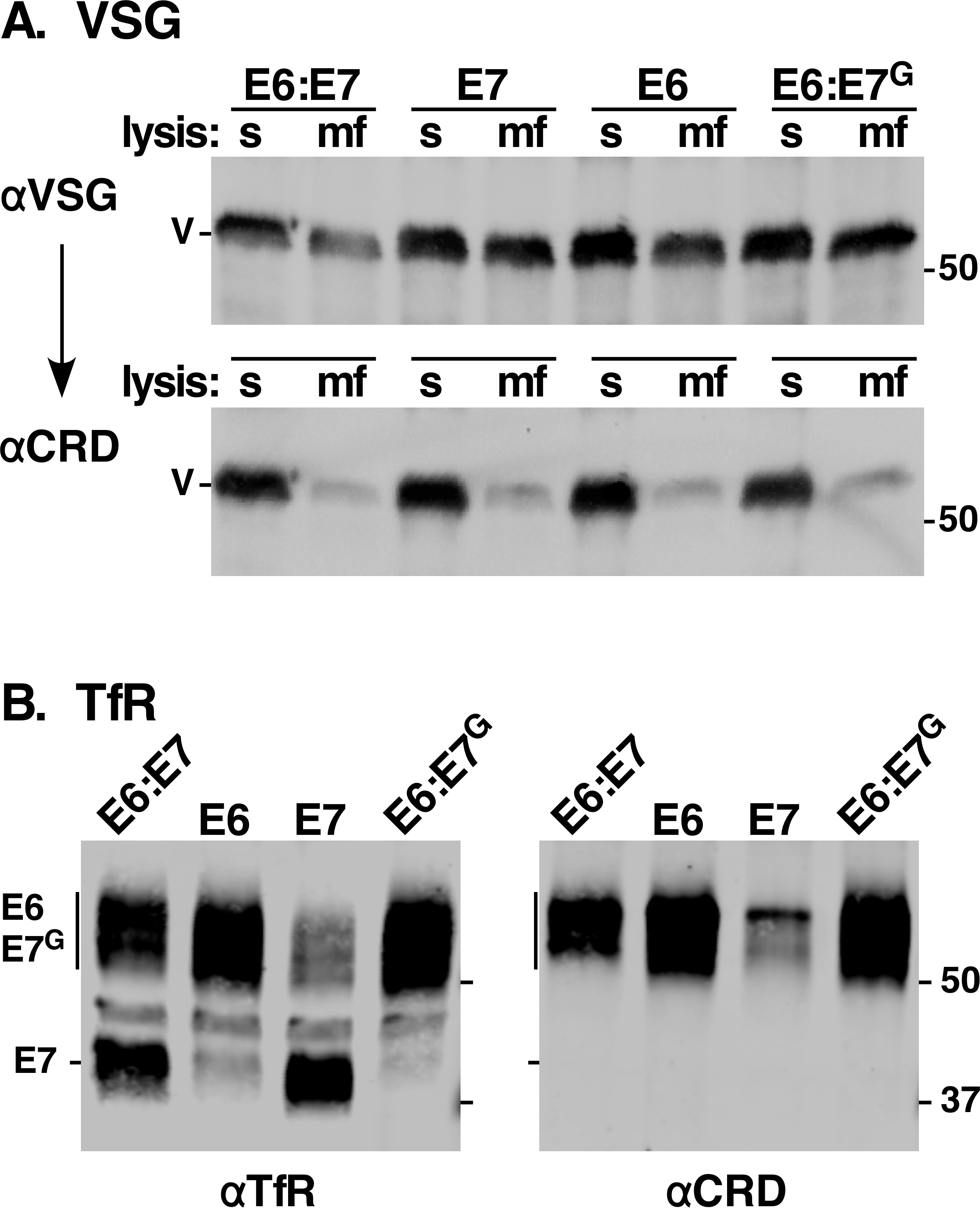

Supplement: S4 Fig — Bloodstream form trypanosomes have an endogenous GPI-specific phospholipase C (GPI-PLC) activity, which is tightly regulated in intact cells, and which has been studied in regard to membrane association of the major GPI-anchored protein, variant surface glycoprotein (VSG) [59, 60]. GPI hydrolysis removes dimyristoylglycerol, leaving behind a 1’, 2’ cyclic inositol monophosphate [17], and converting native VSG from membrane-form (mfVSG) to soluble-form (sVSG) [61] The residual GPI structure on sVSG, but not the intact structure on mfVSG, forms a cross-reacting determinant (CRD) that reacts with specific anti-CRD antibodies present in hyperimmune sera of rabbits immunized with sVSG [62]. Such reactivity is diagnostic for the presence of a GPI anchor, and cell lysates can be prepared in which GPI anchors are all hydrolysed (s-lysis, CRD+) or all intact (mf-lysis, CRD-) [20, 63]. These properties hold for any GPI-anchored protein in BSF trypanosomes, and form the basis for our analyses of the GPI status of our TfR reporters. TfR cell lines were lysed as follows: For s-lysis washed cells were suspended at 1x10^8^ cells/ml in TEN buffer (50 mM TrisHCl, pH 7.5, 150 mM NaCl, 5 mM EDTA) containing 1% NP40 and protease inhibitor cocktail (PIC). Lysates were incubated at 37°C for 5 minutes to allow complete hydrolysis of all GPI anchors, and were then adjusted to final detergent conditions for immunoprecipitation (1x10^7^ cells/ml in TEN containing 1% NP40, 0.5% deoxycholate, 0.1% SDS, PIC). For mf-lysis cells were suspended at 1x10^8^ cells/ml in TEN containing 1% SDS with PIC and boiled for 5 minutes to denature endogenous GPI-PLC. Lysates were cooled and adjusted to final detergent conditions as defined above. All lysates were clarified by centrifugation prior to immunoprecipitation. A. Lysates (s and mf as indicated) from E6R:E7R, E7R, E6R and E6R:E7G RNAiR cell lines (as indicated) were immunoprecipitated with anti-VSG221 antibodies covalently cross-linked to protein A sepharos [file ppat.1006366.s004.tif]
